# Facile and General Synthesis of Photoactivatable Xanthene Dyes**

**DOI:** 10.1002/anie.201104571

**Published:** 2011-09-26

**Authors:** Laura M Wysocki, Jonathan B Grimm, Ariana N Tkachuk, Timothy A Brown, Eric Betzig, Luke D Lavis

**Affiliations:** Janelia Farm Research CampusHoward Hughes Medical Institute 19700 Helix Drive, Ashburn, VA 20147 (USA)

**Keywords:** caged compounds, fluorescence, imaging agents, rhodamine, super-resolution microscopy

## Abstract

**Despite the apparent simplicity:**

of the xanthene fluorophores, the preparation of caged derivatives with free carboxy groups remains a synthetic challenge. A straightforward and flexible strategy for preparing rhodamine and fluorescein derivatives was developed using reduced, “leuco” intermediates.

Photoactivatable “caged” fluorophores enable numerous advanced biological imaging experiments,^[1]^ including photoactivated localization microscopy (PALM)^[2]^ and related super-resolution imaging techniques.^[3]^ Of the extant fluorophore scaffolds, caged rhodamines and fluoresceins display properties that are exceptionally well suited for super-resolution microscopy, exhibiting high contrast and photon yields. The utility of these probes in PALM imaging has been hampered, however, by inefficient syntheses. For example, the existing route to caged Q-rhodamine, a promising PALM probe,^[2]^ requires harsh, strongly basic conditions and is reported to “generate many products” with yields given as “poor” and “variable”.^[1a]^ Such difficult, inefficient syntheses have rendered these important caged molecules unavailable to the scientific community.

We set out to develop a general synthesis of photoactivatable xanthene fluorophores and evaluate these dyes as PALM labels. We recognized the synthetic difficulties of caged xanthenes are inextricably linked with the fluorogenic mechanism of the dyes. Rhodamines and fluoresceins exist in equilibrium between a brightly fluorescent, “open” quinoid structure and a colorless, “closed” lactone.^[1b]^ This equilibrium can be controlled in a light-dependent manner using several strategies^[1,4]^ with the most versatile involving attachment of electron withdrawing photolabile groups to the aniline nitrogens of rhodamine or phenolic oxygens of fluorescein. While the open–closed equilibrium is essential for the fluorogenic properties of the caged dyes, this attribute also complicates their syntheses. The quinoid form of the dye, which predominates under the basic conditions required for functionalization, exhibits poor solubility and low reactivity thereby frustrating the installation of caging groups.

Since the open–closed equilibrium is both the basis for fluorogenicity and the cause of synthetic difficulties, we envisioned eliminating this process in a reversible manner. Rhodamines and fluoresceins can be reduced to “leuco” derivatives, which are widely used sensors for reactive oxygen species^[5]^ but essentially unexplored as synthetic intermediates for fluorogenic derivatives. We surmised reduction of the xanthene core would increase the reactivity of the aniline nitrogens in rhodamines and the phenolic oxygens in fluoresceins allowing installation of caging groups using mild conditions. Here, we establish the use of leuco-dyes as an effective method to prepare caged fluorophores. This efficient and general route enables the preparation of the elusive caged Q-rhodamine (Rh_Q_) with exceptional ease, and can be extended to rhodamine 110 (Rh_110_) and 2′,7′-difluorofluorescein^[6]^ (Oregon Green) derivatives bearing free carboxyl groups for bioconjugation. This synthetic method facilitated the evaluation of these probes for super-resolution microscopy, culminating in the first PALM imaging of DNA in a cellular context.

Our synthesis of a caged Q-rhodamine is shown in Scheme 1. Condensation of trimellitic anhydride (**1**) and 7-hydroxy-1,2,3,4-tetrahydroquinoline (**2**) gave a crude isomeric mixture of 5(6)-carboxy-Rh_Q_.^[1a]^ To install base-labile protecting groups^[7]^ on the nitrogen substitutents we treated this material with trifluoroacetic anhydride (TFAA), affording 5(6)-carboxy-Rh_Q_-bis(trifluoroacetamide). The trifluoroacetamides also facilitated isolation of 5-carboxy-Rh_Q_-bis(trifluoroacetamide) (**3**) by straightforward crystallization. Compound **3** was reduced to the leuco-rhodamine by catalytic hydrogenation at ambient temperature and pressure.^[8]^ Esterification with 2,4-dimethoxybenzyl (DMB) alcohol (**4**) using *N*,*N*′-dicyclohexylcarbodiimide (DCC) and catalytic 4-dimethylaminopyridine (DMAP) furnished leuco-rhodamine diester **5** in 74 % yield over the two-step sequence. Hydrolysis of the trifluoroacetamides, followed by acylation with chloroformate **6** to install the *ortho*-nitroveratryloxycarbonyl (NVOC) cages, yielded reduced rhodamine **7**. The aniline nitrogens in the leuco-Q-rhodamine are more reactive than in Q-rhodamine, allowing high-yielding functionalization under mild conditions. This step represents a significant improvement over the reported reaction to install caging groups onto Rh_Q_.^[1a]^ Treatment of the protected, reduced rhodamine adduct **7** with the mild oxidant 2,3-dichloro-5,6-dicyanobenzoquinone (DDQ) allowed removal of the DMB esters^[9]^ with concomitant oxidation of the reduced dye core. This reaction yields fully deprotected and oxidized NVOC_2_-5-carboxy-Rh_Q_ (**8**).

**Scheme 1:**
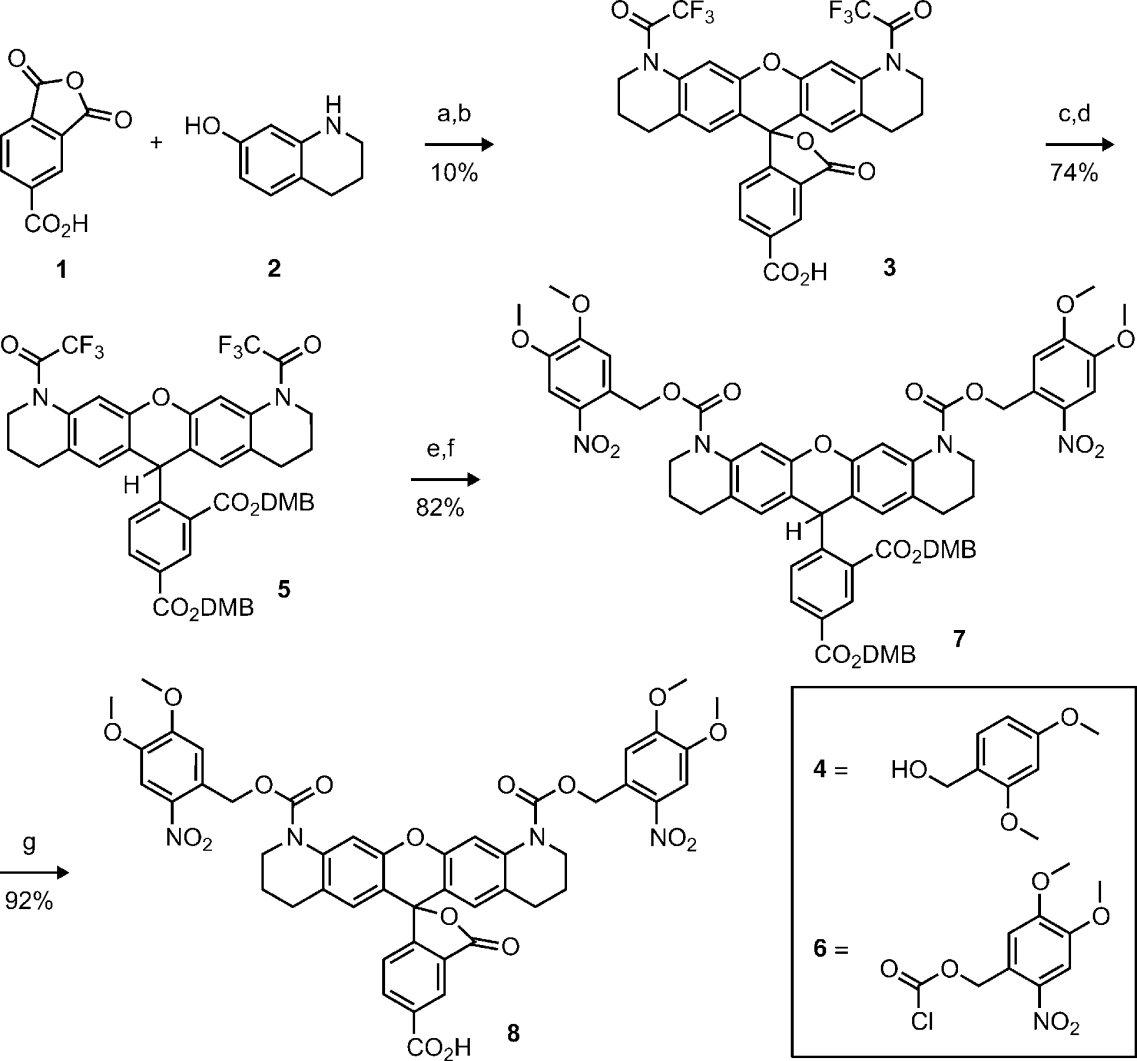
Synthesis of NVOC_2_-5-carboxy-Rh_Q_ 8. a) TsOH, EtCO_2_H, reflux. b) TFAA, py, CH_2_Cl_2_. c) H_2_(g), Pd/C, THF. d) 4, DCC, DMAP, CH_2_Cl_2_. e) NH_4_CO_3_H, THF, H_2_O, CH_3_OH. f) 6, DIEA, CH_2_Cl_2_. g) DDQ, CH_2_Cl_2_ (wet), reflux.

To test the generality of this leuco-dye approach, we applied this strategy to rhodamine 110. Installation of caging groups onto Rh_110_ requires highly reactive electrophiles^[10]^ and the synthesis of caged 5-carboxy-Rh_110_ derivatives has not been reported. Based on our success with Rh_Q_, we first attempted the preparation of 5-carboxy-Rh_110_-bis(trifluoroacetamide) through the reaction of 3-aminophenol and trimellitic anhydride,^[11]^ followed by treatment with TFAA. This protocol delivered a complex mixture of rhodamine and rhodol products that precluded purification by chromatography or crystallization. We therefore developed a novel approach to the preparation of 5-carboxy-Rh_110_ derivatives as shown in Scheme 2. 5-Carboxy-3′,6′-dibromofluoran **9**^[12]^ was protected as the benzyl ester. Pd-catalyzed cross-coupling^[12]^, ^[13]^ of the aryl bromide substituents in **10** with benzophenone imine gave rhodamine **11**. Hydrolysis of this diimine using aqueous acid and subsequent amidation with TFAA afforded bis(trifluoroacetamide) **12** in excellent yield over two steps. Reduction of **12** under H_2_(g) and Pd/C produced leuco-rhodamine 110. The resulting free carboxyl groups were esterified using benzyl alcohol **4**, DMAP, and 3-(3-dimethylaminopropyl)carbodiimide (EDC) to produce diester **13**. Deprotection of the aniline groups with NH_2_OH,^[7]^ followed by acylation with **6** to install the NVOC cages, yielded intermediate **14**. Paralleling the Rh_Q_ example, oxidation of leuco-rhodamine adduct **14** using DDQ gave NVOC_2_-5-carboxy-Rh_110_ **15**.

**Scheme 2:**
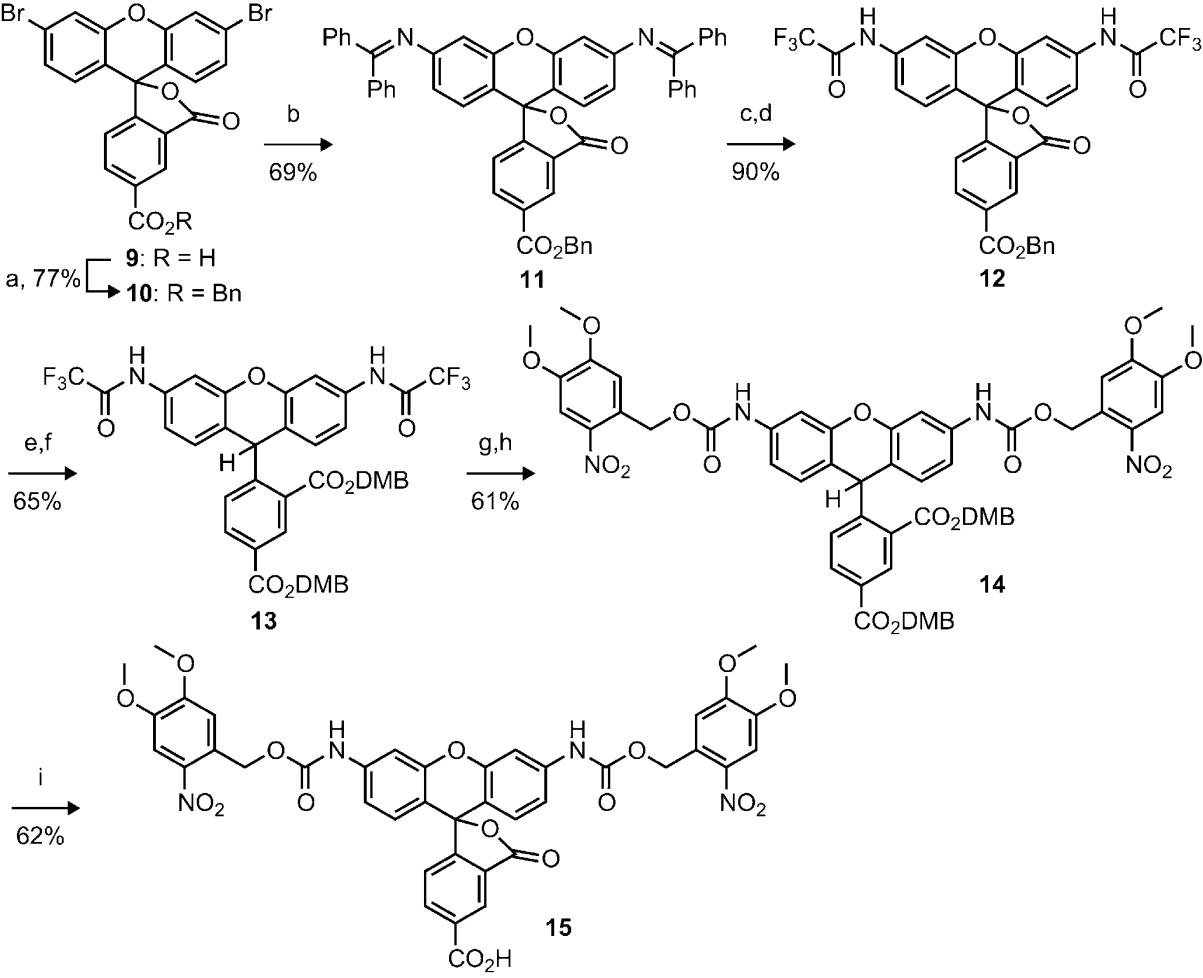
Synthesis of NVOC_2_-5-carboxy-Rh_110_ 15. a) BnOH, EDC, DMAP, CH_2_Cl_2_. b) Pd(OAc)_2_, binap, Cs_2_CO_3_, toluene, 100 °C. c) 5 % HCl/THF. d) TFAA, py, CH_2_Cl_2_. e) H_2_(g), Pd/C, THF. f) 4, EDC, DMAP, CH_2_Cl_2_/EtOAc. g) NH_2_OH, CH_3_OH. h) 6, DIEA, CH_2_Cl_2_. i) DDQ, CH_2_Cl_2_ (wet), reflux.

We also used this approach with a fluorescein dye, the photostable 2′,7′-difluorofluorescein,^[6]^ as shown in Scheme ^[3]^. Fluoresceins are easier to derivatize than rhodamines,^[1a]^ but treatment with alkylating agents gives undesired, fluorescent ether–esters as the major products, due to competing reactivity of the *ortho*-carboxylate. The desired nonfluorescent caged fluorescein is typically obtained in low yield and requires extensive purification.^[14]^ Our synthetic strategy eliminates this unproductive route while improving solubility in organic solvents. 5-Carboxy-2′,7′-difluorofluorescein diacetate (**16**)^[6]^ was reduced to the leuco-fluorescein diacetate by catalytic hydrogenation. Esterification of the resulting diacid with alcohol **4** using *N*,*N*′-diisopropylcarbodiimide (DIC) gave the tetraester **17**. Selective hydrolysis of the acetate esters afforded diphenol **18**, which was efficiently alkylated with bromide **19** using phase-transfer conditions to install the *ortho*-nitroveratryl (NV) photolabile groups in diether **20**. Treatment with DDQ gave the desired caged NV_2_-5-carboxy-2′,7′-difluorofluorescein **21** in good yield, showing the final oxidation step is general for both rhodamine and fluorescein dyes.

**Scheme 3:**
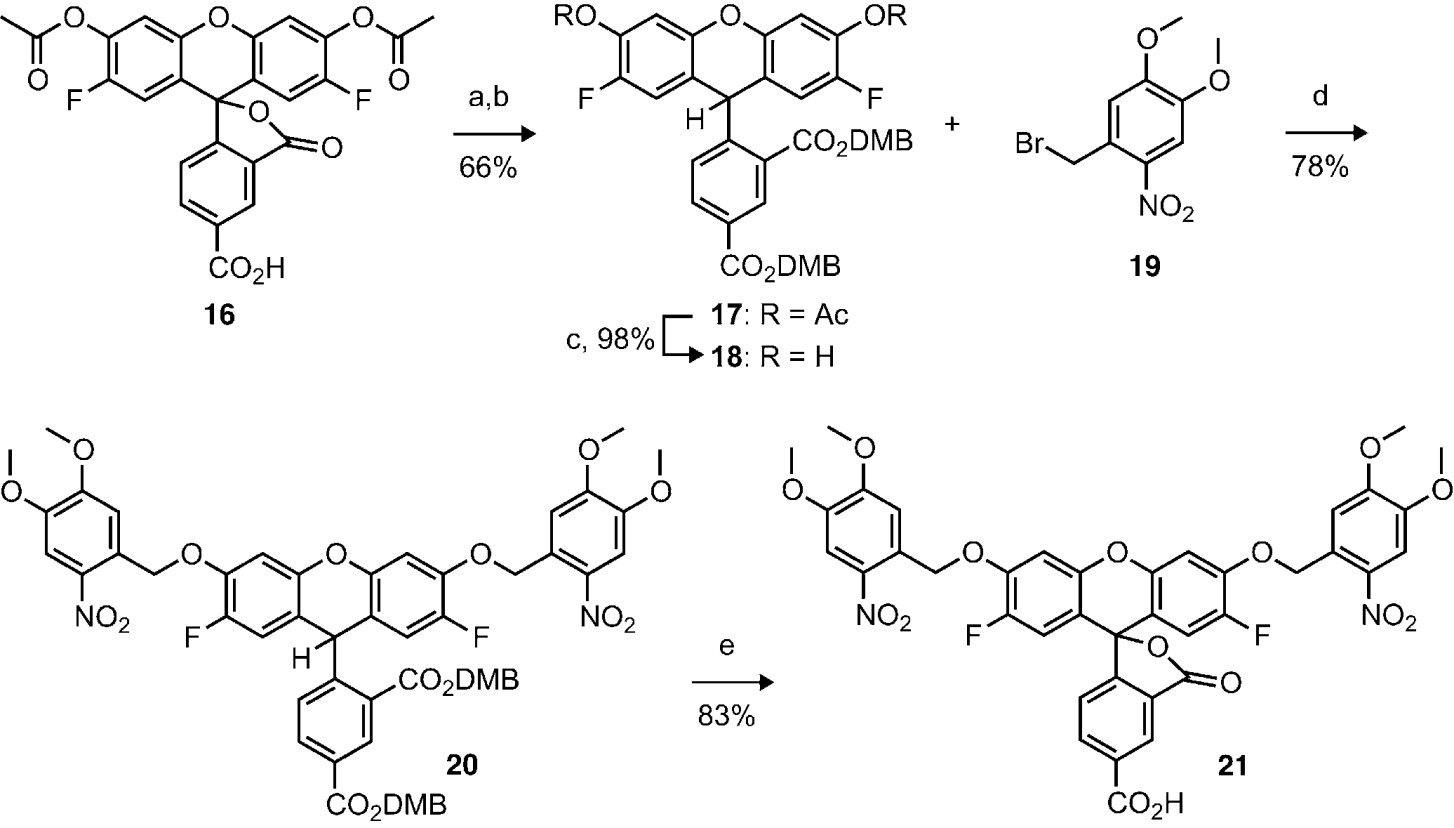
Synthesis of NV_2_-5-carboxy-2′,7′-difluorofluorescein 21. a) H_2_(g), Pd/C, THF. b) 4, DIC, DMAP, CH_2_Cl_2_. c) NH_4_CO_3_H, THF, H_2_O, CH_3_OH. d) NH_4_HSO_4_, K_2_CO_3_, H_2_O, CH_2_Cl_2_. e) DDQ, CH_2_Cl_2_ (wet), reflux.

We then investigated the chemical and photophysical properties of the caged dyes. Biotin conjugates of compounds **8**, **15**, and **21** were synthesized by straightforward amidation of the free carboxyl groups revealed during the final step of the synthesis (see Schemes S1–S3 in the Supporting Information). The chemical stability of these molecules was assessed at pH 5–9; we observed negligible (<1 %) spontaneous uncaging after 48 h (Figure S1). We also determined the average photon yields for the biotin adducts of **8**, **15**, and **21** as well as biotinylated mEos2, a photoswitchable protein that is used widely in PALM imaging and exhibits a localization precision of 11 nm.^[15]^ Both of the caged rhodamines, **8**-biotin and **15**-biotin, showed excellent properties with mean photon yields exceeding or equivalent to mEos2-biotin (139 % and 97 %, respectively; Figure S2). The caged Oregon Green **21**-biotin showed lower photostability than the rhodamine dyes with an average photon yield at 60 % relative to mEos2. Thus, photoactivatable small-molecule fluorophores, especially caged rhodamines, exhibit photon yields that are comparable to an established photoactivatable label capable of high resolution PALM.

Based on the favorable photon yields exhibited by compounds **8**, **15**, and **21** we explored the utility of these dyes in a cellular super-resolution microscopy experiment. Again taking advantage of the free carboxyl groups in these molecules, we prepared azide-containing derivatives of rhodamines **8** and **15** and fluorescein **21** (Schemes S4–S6). This allowed metabolic labeling of cellular DNA by first incubating cells with 5-ethynyl-2′-deoxyuridine (EdU) followed by the Cu^I^-catalyzed Huisgen 1,3-dipolar cycloaddition between the alkynyl nucleobase and the caged dye–azide conjugate (i.e., “click chemistry”).^[16]^ This labeling allowed super-resolution localization microscopy of labeled cellular DNA using all three dyes as shown in Figure 1. Diffraction limited summed total internal reflection fluorescence (TIRF) microscopy images are also given for comparison. As expected from previous in vitro experiments,^[2]^ the Rh_Q_ derivative **8**-azide was a viable probe for PALM imaging (Figure 1a). Of the shorter-wavelength probes, the caged Rh_110_ **15**-azide derivative (Figure 1b) gave better PALM images than fluorescein **21**-azide (Figure 1c) due to the higher photon yield and lower nonspecific staining. While super-resolution microscopy of purified DNA has been performed by direct stochastic optical reconstruction microscopy (dSTORM)^[17]^ and stimulated emission depletion (STED) microscopy,^[18]^ this is the first example of super-resolution microscopy of DNA in a cellular context. Moreover, the PALM-caged fluorophore system circumvents the redox buffer conditions necessary for dSTORM and the sculpted light requirement of STED. Molecular maps of cellular DNA will enable precise identification of DNA–protein interactions and determination of the location of these complexes within the cell.^[19]^

**Figure 1:**
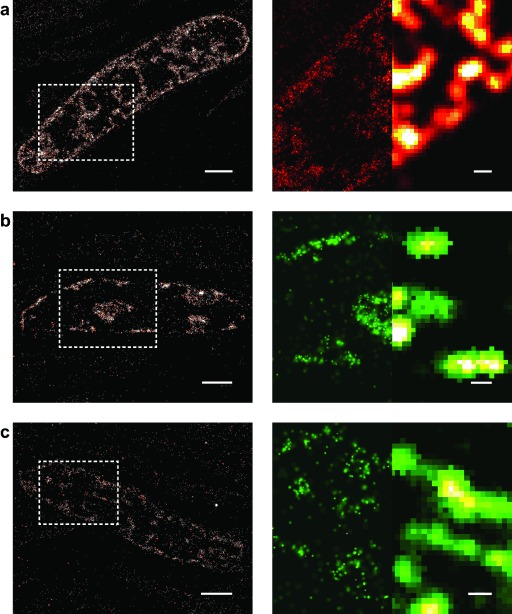
PALM images of nuclei from fixed, cryosectioned 3T3 cells labeled with photoactivatable dyes. Left panel: molecule localization image; scale bar: 2 μm. Right panel: zoomed localization probability image/summed-TIRF image; scale bar: 500 nm. a)  8 -azide; b)  15 -azide; c)  21 -azide.

In summary, we have established a facile and general strategy to prepare caged rhodamine and fluorescein photoactivatable labels for super-resolution microscopy. The use of leuco-dye intermediates overcomes difficulties with reactivity and solubility, allowing the preparation of these valuable molecules with reaction conditions accessible to any organic chemistry laboratory. Our efficient final synthetic step utilizes a mild oxidant to concurrently remove protecting groups and oxidize the dye. We note this caged xanthene system is highly modular, allowing independent tuning of the dye and the cage. While the caged Rh_Q_ and Rh_110_ dyes already constitute a potential pair of dyes for multicolor PALM imaging, our divergent synthetic route will enable the synthesis of xanthene and isologous^[20]^ dyes of different wavelengths bearing cages with tailored chemical and optical properties. These efforts will create a palette of probes to bolster the diminutive collection of synthetic photoactivatable fluorescent labels suitable for super-resolution imaging experiments.^[1c]^, ^[4]^, ^[21]^ Finally, this facile leuco-dye strategy is appropriate for the installation of other blocking groups and will allow the construction of a wide range of useful fluorogenic dyes.

## Notes

Detailed facts of importance to specialist readers are published as ”Supporting Information”. Such documents are peer-reviewed, but not copy-edited or typeset. They are made available as submitted by the authors.

## References

[b1a] Mitchison TJ, Sawin KE, Theriot JA, Gee K, Mallavarapu A, Marriott G (1998). Methods Enzymol.

[b1b] Lavis LD, Raines RT (2008). ACS Chem. Biol.

[b1c] Fernández-Suárez M, Ting A (2008). Nat. Rev. Mol. Cell Biol.

[b1d] Puliti D, Warther D, Orange C, Specht A, Goeldner M (2011). Bioorg. Med. Chem.

[2] Betzig E, Patterson GH, Sougrat R, Lindwasser OW, Olenych S, Bonifacino JS, Davidson MW, Lippincott-Schwartz J, Hess HF (2006). Science.

[b3] Hess ST, Girirajan TPK, Mason MD (2006). Biophys. J.

[b3b] Rust MJ, Bates M, Zhuang X (2006). Nat. Methods.

[b4] Fölling J, Belov V, Riedel D, Schonle A, Egner A, Eggeling C, Bossi M, Hell SW (2008). ChemPhysChem.

[b4b] Belov VN, Wurm CA, Boyarskiy VP, Jakobs S, Hell SW Angew. Chem.

[5] Royall JA, Ischiropoulos H (1993). Arch. Biochem. Biophys.

[6] Sun W-C, Gee KR, Klaubert DH, Haugland RP (1997). J. Org. Chem.

[7] Haugland RP, Spence MTZ, Johnson ID (1996). Handbook of Fluorescent Probes and Research Chemicals.

[8] Zaikova TO, Rukavishnikov AV, Birrell GB, Griffith OH, Keana JFW (2001). Bioconjugate Chem.

[9] Kim C, Misco P (1985). Tetrahedron Lett.

[10] Ottl J, Gabriel D, Marriott G (1998). Bioconjugate Chem.

[11] Ioffe IS, Otten VF (1961). Zh. Obshch. Khim.

[12] Woodroofe CC, Lim MH, Bu WM, Lippard SJ (2005). Tetrahedron.

[13] Jin X, Uttamapinant C, Ting AY (2011). ChemBioChem.

[b14] Krafft GA, Sutton WR, Cummings RT (1988). J. Am. Chem. Soc.

[b14b] Mitchison TJ (1989). J. Cell Biol.

[15] McKinney SA, Murphy CS, Hazelwood KL, Davidson MW, Looger LL (2009). Nat. Methods.

[16] Salic A, Mitchison TJ (2008). Proc. Natl. Acad. Sci. USA.

[17] Flors C, Ravarani CNJ, Dryden D (2009). ChemPhysChem.

[18] Persson F, Bingen P, Staudt T, Engelhardt J, Tegenfeldt J, Hell SW Angew. Chem.

[19] Yao J, Fetter RD, Hu P, Betzig E, Tjian R (2011). Genes Dev.

[20] Kolmakov K, Belov VN, Bierwagen J, Ringemann C, Müller V, Eggeling C, Hell SW (2010). Chem. Eur. J.

[b21] Dempsey GT, Bates M, Kowtoniuk WE, Liu DR, Tsien RY, Zhuang X (2009). J. Am. Chem. Soc.

[b21b] Lee HD, Lord SJ, Iwanaga S, Zhan K, Xie H, Williams JC, Wang H, Bowman GR, Goley ED, Shapiro L, Twieg RJ, Rao J, Moerner WE (2010). J. Am. Chem. Soc.

