# Supplementary material for: Facile and General Synthesis of Photoactivatable Xanthene Dyes
Source: Angew Chem Int Ed Engl. 2011 Sep 26;50(47):11206–9. doi: 10.1002/anie.201104571 (PMC3588110; doi:10.1002/anie.201104571)
Supplement: Supplementary file 1 [file anie0050-11206-sd1.pdf]

Angewandte Chemie
Eine Zeitschrift der Gesellschaft Deutscher Chemiker

Supporting Information
© Wiley-VCH 2011
69451 Weinheim, Germany

Facile and General Synthesis of Photoactivatable Xanthene Dyes**

*Laura M. Wysocki, Jonathan B. Grimm, Ariana N. Tkachuk, Timothy A. Brown, Eric Betzig, and Luke D. Lavis**

anie_201104571_sm_miscellaneous_information.pdf

SYNTHETIC METHODS

General Chemical Synthesis Methods. Compound **2** was from Allichem. Compound **16** was synthesized as previously described.[1] Compound **S2** was from Pierce. Compound **S7** was from Quanta BioDesign. TSTU was from TCI America. All other reagents were the highest grade available and obtained from Sigma–Aldrich or Fisher Scientific. Reactions were stirred under N_2(g) unless otherwise noted. Anhydrous solvents were drawn from Aldrich Sure-Seal bottles or from a column-based solvent purification system from Innovative Technology. Thin-layer chromatography was performed by using aluminum-backed plates coated with silica gel containing F_{254} phosphor and visualized by UV-illumination or charring.

Flash chromatography was performed on an Isolera 4 system with SNAP columns (Biotage). The term "concentrated *in vacuo*" refers to the removal of solvents and other volatile materials by using a Büchi Rotavapor at variable pressure (controlled diaphragm pump; ≥1 mm Hg) while maintaining the water bath temperature below 40 °C or on a Thermo Fisher Explorer SpeedVac Concentrator at variable pressure (controlled diaphragm pump; ≥0.5 mm Hg) while maintaining the chamber temperature below 40 °C.

Preparative HPLC was performed with an Agilent 1200 HPLC system equipped with a G1315D diode array detector, and a G1364B fraction collector using a 30 × 150 mm XTerra Prep MS C18 OBD (10 μm) column. Tandem HPLC–mass spectrometry (LC–MS) was performed on Agilent 1200 HPLC system equipped with a G1315B diode array detector and a 6130 mass spectrometry detector using 4.6 × 150 mm XTerra MS C18 (5 μm) column. Mass spectrometry was performed by the Mass Spectrometry Center in the Department of Medicinal Chemistry at the University of Washington. NMR spectra were obtained with a Bruker 400 MHz Avance-II$^+$ spectrometer at the Janelia Farm Research Campus. ^{1}H and ^{13}C NMR spectra were referenced to TMS or residual solvent peaks. ^{19}F NMR spectra were referenced to $CFCl_3$.

3-Oxo-1',11'-bis(2,2,2-trifluoroacetyl)-1',2',3',4',8',9',10',11'-octahydro-3*H*-spiro[isobenzofuran-1,6'-pyrano[3,2-*g*:5,6-*g'*]di-quinoline]-5-carboxylic acid (3; *i.e.*, 5-carboxy-Q-rhodamine bis(trifluoroacetamide)): Trimellitic anhydride (**1**, 644 mg, 3.35 mmol, 1.0 eq) and *p*-toluenesulfonic acid monohydrate (128 mg, 0.670 mmol, 0.2 eq) were added to a solution of 7-hydroxy-1,2,3,4-tetrahydroquinoline (**2**, 1.0 g, 6.70 mmol, 2.0 eq) in 8 mL of propionic acid. The mixture was heated to reflux, stirred for 3 d, and cooled to room temperature. The reaction was poured into 200 mL of 5% v/v aqueous HCl while stirring. The dark red precipitate was collected via filtration and dried.

A solution of the crude dark red solid in 6 mL CH_2Cl_2 was cooled to 0 °C and trifluoroacetic anhydride (1.44 mL, 10.4 mmol, 4.0 eq) and pyridine (840 μL, 10.4 mmol, 4.0 eq) were carefully added. The ice bath was removed and the reaction was warmed to room temperature and stirred for 2 h. The reaction was diluted to 30 mL CH_2Cl_2 and quenched with 15 mL of 10% w/v aqueous citric acid. The layers were partitioned and the aqueous layer was extracted with CH_2Cl_2 (2×30 mL). The organic layers were combined, dried over anhydrous $MgSO_4$(s), filtered, and concentrated *in vacuo*. Purification by column chromatography (CH_2Cl_2→4:1 v/v CH_2Cl_2/EtOAc, linear gradient, with constant 1% v/v AcOH additive) afforded a mixture of the 5- and 6-carboxy-Q-rhodamine bis(trifluoroacetamide) isomers as a pale pink solid. The two isomers were separated by successive crystallization of this mixture from boiling EtOAc/hexanes. The initial crop of crystals proved to be 6-carboxy-Q-rhodamine bis(trifluoroacetamide) as determined by ^{1}H NMR.[2] The mother liquor was concentrated *in vacuo* and crude **3** was recrystallized from boiling EtOAc/hexanes to yield the desired 5-carboxy-Q-rhodamine **3** (210.8 mg, 10% yield, 2 steps, >95% purity).

Data for **3**: **R$_f$** = 0.48 (8:2:0.1 v/v/v CH_2Cl_2/CH_3CN/AcOH); **^{1}H NMR** (400 MHz, $CDCl_3$, δ): 2.03 (m, 4H), 2.66 (ABXY, J_{AB} = 16.5 Hz, J_{AX} = 6.5 Hz, J_{AY} = 6.5 Hz, 2H), 2.75 (ABXY, J_{AB} = 16.5 Hz, J_{BX} = 7.4 Hz, J_{BY} = 7.4 Hz, 2H), 3.74 (ABXY, J_{AB} = 13.3 Hz, J_{AX} = 6.1 Hz, J_{AY} = 6.1 Hz, 2H), 3.93 (ABXY, J_{AB} = 13.3 Hz, J_{BX} = 5.6 Hz, J_{BY} = 5.6 Hz, 2H), 6.64 (s, 2H), 7.28 (dd, *J* = 8.1, 0.6 Hz, 1H), 7.75 (br s, 2H), 8.41 (dd, *J* = 8.1, 1.5 Hz, 1H), 8.79 (dd, *J* = 1.5, 0.6 Hz, 1H); **^{13}C NMR** (100 MHz, $CDCl_3$, δ): 23.0 (CH_2), 25.5 (CH_2), 45.0 (q, $^4J_{CF}$ = 3.3 Hz, CH_2), 81.8 (C), 113.2 (CH), 116.0 (C), 116.5 (q, $^1J_{CF}$ = 288 Hz, C), 124.3 (CH), 126.1 (C), 127.5 (CH), 127.7 (CH), 131.6 (C), 136.9 (CH), 138.9 (C), 148.9 (C), 156.0 (q, $^2J_{CF}$ = 35.6 Hz, C), 158.0 (C), 168.3 (C), 169.0 (C); **^{19}F NMR** (376 MHz, $CDCl_3$, δ): –69.1 (br s); **HRMS** (*m/z*): $[M+H]^+$ calculated, 647.1247; observed 647.1236.

Data for **6-carboxy-Q-rhodamine bis(trifluoroacetamide)**: **R$_f$** = 0.48 (8:2:0.1 v/v/v CH_2Cl_2/CH_3CN/AcOH); **^{1}H NMR** (400 MHz, $CDCl_3$, δ): 2.02 (m, 4H), 2.65 (ABXY, J_{AB} = 16.6 Hz, J_{AX} = 6.9 Hz, J_{AY} = 6.9 Hz, 2H), 2.72 (ABXY, J_{AB} = 16.6 Hz, J_{BX} = 7.4 Hz, J_{BY} = 7.4 Hz, 2H), 3.76 (ABXY, J_{AB} = 13.3 Hz, J_{AX} = 6.1 Hz, J_{AY} = 6.1 Hz, 2H), 3.90 (ABXY, J_{AB} = 13.3 Hz, J_{BX} = 5.7 Hz, J_{BY} = 5.7 Hz, 2H), 6.59 (s, 2H), 7.74 (br s, 2H), 7.82 (s, 1H), 8.14 (dd, *J* = 8.0, 0.6 Hz, 1H), 8.34 (dd, *J* = 8.0, 1.4 Hz, 1H); **^{13}C NMR** (100 MHz, $CDCl_3$, δ): 23.1 (CH_2), 25.6 (CH_2), 45.0 (q, $^4J_{CF}$ = 2.2 Hz, CH_2), 82.0 (C), 113.2 (CH), 116.0 (C), 116.5 (q, $^1J_{CF}$ = 288 Hz, C), 125.6 (CH), 125.7 (C), 127.6 (CH), 129.7 (C), 131.8 (CH), 135.7 (C), 138.9 (C), 149.1 (C), 153.7 (C), 156.1 (q, $^2J_{CF}$ = 36.3 Hz, C), 168.1 (C), 168.3 (C); **^{19}F NMR** (376 MHz, $CDCl_3$, δ): –69.1 (br s); **HRMS** (*m/z*): $[M+Na]^+$ calculated, 669.1067; observed 669.1058.

Bis(2,4-dimethoxybenzyl) 4-(1,11-bis(2,2,2-trifluoroacetyl)-2,3,4,6,8,9,10,11-octahydro-1*H*-pyrano[3,2-*g*:5,6-*g'*]diquinolin-6-yl)isophthalate (5): To a solution of 5-carboxy-Q-rhodamine derivative **3** (140. mg, 0.217 mmol) in 6 mL THF was added 10% w/w palladium on carbon (14 mg). The reaction flask was flushed thoroughly with nitrogen gas, followed by hydrogen gas. The reaction was stirred for 16 h at room temperature under 1 atm of hydrogen gas (balloon), after which time, the reaction flask was thoroughly purged with nitrogen gas. The crude reaction mixture was filtered though celite, rinsed with THF (30 mL), and concentrated *in vacuo*, affording a clear oil that turns pink upon prolonged exposure to air.

To a solution of the crude reduced Q-rhodamine derivative in 7 mL CH_2Cl_2 was added *N,N'*-dicyclohexylcarbodiimide (DCC, 196.6 mg, 0.953 mmol, 4.4 eq), 4-dimethylaminopyridine (DMAP, 0.5 mg, 4.3 μmol, 0.02 eq), and 2,4-dimethoxybenzyl alcohol (**4**, 160.3 mg, 0.953 mmol, 4.4 eq). The reaction was stirred at room temperature for 18 h. The mixture was deposited onto celite and purified by column chromatography (hexanes→3:1 v/v EtOAc/hexanes, linear gradient, with constant 40% v/v CH_2Cl_2 additive), affording 151.2 mg of pure diester **5** as a white foamy solid (74% yield, 2 steps).

Data for **5**: **R$_f$** = 0.36 (8:2:0.1 v/v/v CH_2Cl_2/CH_3CN/AcOH); **^{1}H NMR** (400 MHz, $CDCl_3$, δ): 1.95 (m, 4H), 2.55 (br s, 4H), 3.63 (s, 3H), 3.78 (m, 4H), 3.79 (s, 6H), 3.81 (s, 3H), 5.29 (s, 2H), 5.41 (s, 2H), 6.19 (s, 1H), 6.37 (d, *J* = 2.2 Hz, 1H), 6.46 (m, 3H), 6.68 (s, 2H), 7.12 (d, *J* = 8.3 Hz, 1H), 7.27 (d, *J* = 8.7 Hz, 1H), 7.33 (d, *J* = 8.3 Hz, 1H), 7.57 (br s, 2H), 7.94 (dd, *J* = 8.3, 1.7 Hz, 1H), 8.44 (d, *J* = 1.6 Hz, 1H); **^{13}C NMR** (100 MHz, $CDCl_3$, δ): 23.2 (CH_2), 25.3 (CH_2), 38.3 (CH), 45.0 (q, $^4J_{CF}$ = 2.6 Hz, CH_2), 55.3 (CH_3), 55.4 (CH_3), 55.4 (CH_3), 55.5 (CH_3), 62.4 (CH_2), 63.6 (CH_2), 98.4 (CH),

98.6 (CH), 104.0 (CH), 104.1 (CH), 112.3 (CH), 115.9 (C), 116.5 (C), 116.6 (q, $^1J_{CF}$ = 288 Hz, C), 122.1 (C), 129.0 (C), 129.8 (CH), 130.6 (C), 130.8 (CH), 131.4 (CH), 131.7 (CH), 132.0 (CH), 133.0 (CH), 136.3 (C), 148.6 (C), 150.9 (C), 156.5 (q, $^2J_{CF}$ = 36.6 Hz, C), 159.1 (C), 159.3 (C), 161.4 (C), 161.8 (C), 165.5 (C), 168.0 (C); **^{19}F NMR** (376 MHz, $CDCl_3$, δ): –69.3 (br s); **HRMS** (*m/z*): $[M+Na]^+$ calculated, 971.2585; observed, 971.2580.

Bis(4,5-dimethoxy-2-nitrobenzyl) 6-(2,4-bis(((2,4-dimethoxybenzyl)oxy)carbonyl)phenyl)-3,4,6,8,9,10-hexahydro-1*H*-pyrano[3,2-*g*:5,6-*g'*]diquinoline-1,11(2*H*)-dicarboxylate (7): To a solution of diester **5** (55.0 mg, 0.0580 mmol) in 2 mL THF and 1 mL MeOH was added ammonium bicarbonate (137 mg, 1.74 mmol, 30 eq) as a solution in 1 mL H_2O. The reaction was stirred vigorously (~600 RPM) at room temperature for 40 h and shielded from light. The organic solvents were concentrated *in vacuo*, the aqueous layer was diluted to 50 mL of saturated $NaHCO_3$, and it was extracted with CH_2Cl_2 (3×50 mL). The organic layers were combined, dried over anhydrous $MgSO_4$(s), filtered, and concentrated *in vacuo* to give the crude material as a pink solid.

To a solution of the crude intermediate dianiline in 2 mL CH_2Cl_2 was added 4,5-dimethoxy-2-nitrobenzyl chloroformate (**6**, 32.0 mg, 0.116 mmol, 2 eq) and *N,N'*-diisopropylethylamine (DIEA, 22 μL, 0.128 mmol, 2.2 eq). The reaction was stirred for 18 h at room temperature while shielded from light, concentrated *in vacuo* and loaded directly onto a column. Purification by column chromatography (hexanes→3:2 v/v hexanes/EtOAc, linear gradient, with constant 40% v/v CH_2Cl_2 additive) afforded pure caged **7** as a white solid (59.0 mg, 82% yield, 2 steps).

Data for **7**: **R_f** = 0.29 (6:2:2 v/v/v hexanes/EtOAc/CH_2Cl_2); **^{1}H NMR** (400 MHz, $CDCl_3$, δ): 1.85 (m, 4H), 2.49 (m, 4H), 3.66 (s, 3H), 3.75 (m, 4H), 3.79 (s, 3H), 3.80 (s, 3H), 3.81 (s, 3H), 3.86 (s, 6H), 3.94 (s, 6H), 5.28 (s, 2H), 5.41 (s, 2H), 5.65 (ABq, J_{AB} = 15.7 Hz, 2H), 5.67 (ABq, J_{AB} = 15.7 Hz, 2H), 6.14 (s, 1H), 6.39 (d, *J* = 2.3 Hz, 1H), 6.45 (m, 3H), 6.63 (s, 2H), 7.04 (s, 2H), 7.08 (d, *J* = 8.3 Hz, 1H), 7.26 (d, *J* = 8.8 Hz, 1H), 7.34 (d, *J* = 8.3 Hz, 1H), 7.52 (br s, 2H), 7.71 (s, 2H), 7.91 (dd, *J* = 8.3, 1.8 Hz, 1H), 8.42 (d, *J* = 1.8 Hz, 1H); **^{13}C NMR** (100 MHz, $CDCl_3$, δ): 23.2 (CH_2), 26.6 (CH_2), 37.9 (CH), 45.0 (CH_2), 55.3 (CH_3), 55.4 (CH_3), 55.5 (CH_3), 56.3 (CH_3), 56.4 (CH_3), 62.4 (CH_2), 63.5 (CH_2), 64.8 (CH_2), 98.5 (CH), 98.6 (CH), 104.0 (CH), 104.1 (CH), 108.2 (CH), 110.4 (CH), 111.3 (CH), 116.1 (C), 116.5 (C), 119.6 (C), 125.3 (C), 127.6 (C), 128.7 (C), 129.4 (CH), 130.4 (C), 130.8 (CH), 131.3 (CH), 131.6 (CH), 131.9 (CH), 132.9 (CH), 137.3 (C), 139.9 (C), 148.1 (C), 148.7 (C), 151.7 (C), 153.5 (C), 154.0 (C), 159.0 (C), 159.3 (C), 161.3 (C), 161.7 (C), 165.5 (C), 168.0 (C); **HRMS** (*m/z*): $[M+Na]^+$ calculated, 1257.3799; observed, 1257.3781.

1',11'-bis(((4,5-dimethoxy-2-nitrobenzyl)oxy)carbonyl)-3-oxo-1',2',3',4',8',9',10',11'-octahydro-3*H*-spiro[isobenzofuran-1,6'-pyrano[3,2-*g*:5,6-*g'*]diquinoline]-5-carboxylic acid (8; *i.e.*, NVOC$_2$-5-carboxy-Q-rhodamine): To a solution of diester **7** (38.9 mg, 0.0315 mmol) in 5.4 mL CH_2Cl_2 and 0.6 mL pH 7 buffer was added 2,3-dichloro-5,6-dicyano-1,4-benzoquinone (DDQ, 71.5 mg, 0.315 mmol, 10 eq). The reaction was heated to reflux and stirred vigorously (~600 RPM) for 24 h while shielded from light. The resulting mixture was cooled to room temperature, filtered through a pad of celite, and concentrated *in vacuo*. Purification by column chromatography (CH_2Cl_2→4:1 v/v CH_2Cl_2/acetone, linear gradient, with constant 1% v/v AcOH additive, column run twice) afforded 26.9 mg (92% yield) of pure NVOC$_2$-5-carboxy-Q-rhodamine **8** as a white solid.

Data for **8**: **R_f** = 0.40 (8:2:0.1 v/v/v CH_2Cl_2/acetone/AcOH); **^{1}H NMR** (400 MHz, $CDCl_3$, δ): 1.90 (m, 4H), 2.56 (ABXY, J_{AB} = 16.0 Hz, J_{AX} = 6.5 Hz, J_{AY} = 6.5 Hz, 2H), 2.63 (ABXY, J_{AB} = 16.0 Hz, J_{BX} = 6.4 Hz, J_{BY} = 6.4 Hz, 2H), 3.80 (m, 4H), 3.91 (s, 6H), 3.96 (s, 6H), 5.66 (ABq, J_{AB} = 14.6 Hz, 2H), 5.69 (ABq, J_{AB} = 14.6 Hz, 2H), 6.49 (s, 2H), 7.04 (s, 2H), 7.26 (d, *J* = 7.8 Hz, 1H), 7.73 (s, 2H), 7.79 (br s, 2H), 8.40 (dd, *J* = 8.0, 0.9 Hz, 1H), 8.78 (s, 1H); **^{13}C NMR** (100 MHz, $CDCl_3$, δ): 22.9 (CH_2), 26.9 (CH_2), 45.2 (CH_2), 56.3 (CH_3), 56.4 (CH_3), 65.0 (CH_2), 82.8 (C), 108.3 (CH), 110.8 (CH), 111.7 (CH), 113.1 (C), 124.4 (CH), 126.2 (C), 126.8 (C), 127.0 (C), 127.1 (CH), 127.5 (CH), 128.2 (C), 129.0 (C), 136.6 (CH), 140.0 (C), 140.1 (C), 148.3 (C), 149.4 (C), 153.5 (C), 154.0 (C), 157.8 (C), 168.3 (C); **HRMS** (*m/z*): $[M+H]^+$ calculated, 933.2461; observed, 933.2473.

Benzyl 3',6'-dibromo-3-oxo-3*H*-spiro[isobenzofuran-1,9'-xanthene]-5-carboxylate (10): To a suspension of 3',6'-dibromo-3-oxo-3H-spiro[isobenzofuran-1,9'-xanthene]-5-carboxylic acid (1.50 g, 2.99 mmol) in CH_2Cl_2 (25 mL) were added benzyl alcohol (404 mg, 3.73 mmol, 1.25 eq), EDCI (860 mg, 4.49 mmol, 1.5 eq), and DMAP (37 mg, 0.299 mmol, 0.1 eq). The reaction was stirred at room temperature overnight, during which time the starting material dissolved to give a yellow solution. It was subsequently diluted with water and extracted with CH_2Cl_2 (2×). The combined organic extracts were washed with brine, dried ($MgSO_4$), filtered, and concentrated *in vacuo* to provide a yellow solid. The solid was triturated with EtOAc/hexanes, filtered, and dried to provide **10** as an off-white solid (1.134 g). The filtrate was concentrated to a yellow residue and purified by silica gel chromatography (0→10% EtOAc/hexanes, linear gradient, with constant 40% v/v CH_2Cl_2 additive) to obtain additional **10** (235 mg). The two crops were combined to afford a combined yield of 1.369 g (77%) of **10**.

Data for **10**: **R_f** = 0.31 (9:1 v/v hexanes/EtOAc); **^{1}H NMR** (400 MHz, $CDCl_3$, δ): 5.42 (s, 2H), 6.67 (d, *J* = 8.5 Hz, 2H), 7.22–7.16 (m, 3H), 7.44–7.34 (m, 3H), 7.48–7.44 (m, 2H), 7.50 (d, *J* = 1.9 Hz, 2H), 8.36 (dd, *J* = 8.0, 1.5 Hz, 1H), 8.71 (dd, *J* = 1.4, 0.7 Hz, 1H); **^{13}C NMR** (100 MHz, $CDCl_3$, δ): 67.8 (CH_2), 81.4 (C), 117.3 (C), 120.7 (CH), 124.1 (CH), 124.7 (C), 126.4 (C), 127.3 (CH), 127.8 (CH), 128.6 (CH), 128.8 (CH), 128.9 (CH), 129.2 (CH), 132.9 (C), 135.4 (C), 136.7 (CH), 151.2 (C), 156.6 (C), 164.7 (C), 168.0 (C); **HRMS** (*m/z*): $[M+H]^+$ calculated, 590.9437; observed, 590.9425.

Benzyl 3',6'-bis((diphenylmethylene)amino)-3-oxo-3H-spiro[isobenzofuran-1,9'-xanthene]-5-carboxylate (11): Dibromide **10** (950 mg, 1.604 mmol), $Pd(OAc)_2$ (72 mg, 0.321 mmol, 0.2 eq), BINAP (300 mg, 0.481 mmol, 0.3 eq), and Cs_2CO_3 (1.463 g, 4.491 mmol, 2.8 eq) were combined in an oven-dried flask; the flask was then sealed and evacuated/backfilled with nitrogen (3×). Toluene (10 mL) and benzophenone imine (644 μL, 3.850 mmol, 2.4 eq) were added, and the reaction vessel was again evacuated/backfilled with nitrogen (3×). The sealed reaction was stirred at 100 °C for 18 h. After cooling to room temperature, the reaction was diluted with CH_2Cl_2, filtered through celite, and concentrated *in vacuo*. Purification by flash chromatography (0→25% EtOAc/hexanes, linear gradient, with constant 1% v/v triethylamine additive) afforded **11** as a yellow foam (876 mg, 69%).

Data for **11**: **R_f** = 0.39 (75:25:1 v/v/v hexanes/EtOAc/Et_3N); **^{1}H NMR** (400 MHz, $CDCl_3$, δ): 5.41 (s, 2H), 6.35 (dd, *J* = 8.4, 2.0 Hz, 2H), 6.49 (d, *J* = 8.4 Hz, 2H), 6.61 (d, *J* = 2.0 Hz, 2H), 7.15–7.06 (m, 5H), 7.32–7.22 (m, 6H), 7.50–7.34 (m, 11H), 7.72 (d, *J* = 7.3 Hz, 4H), 8.31 (dd, *J* = 8.1, 1.5 Hz, 1H), 8.63 (dd, *J* = 1.4, 0.7 Hz, 1H); **^{13}C NMR** (100 MHz, $CDCl_3$, δ): 67.6 (CH_2), 83.3 (C), 108.8 (CH), 112.6 (C), 117.1 (CH), 124.3 (CH), 126.7 (CH), 127.0 (C), 128.2 (CH), 128.3 (CH), 128.4 (CH), 128.6 (CH), 128.7 (CH), 128.9 (CH), 129.2 (CH), 129.4 (CH), 129.6 (CH), 131.2 (CH), 132.2 (C), 135.5 (C), 135.6 (C), 136.2 (CH), 139.3 (C), 151.6 (C), 153.9 (C),

157.6 (C), 165.0 (C), 168.7 (C), 169.3 (C); **HRMS** (*m/z*): $[M+H]^+$ calculated, 793.2697; observed, 793.2678.

Benzyl 3-oxo-3',6'-bis(2,2,2-trifluoroacetamido)-3H-spiro[isobenzofuran-1,9'-xanthene]-5-carboxylate (12): Diimine **11** (849 mg, 1.071 mmol) was taken up in THF (7.5 mL), and 5% HCl (7.5 mL) was added. The reaction, which immediately turned red, was stirred at room temperature for 3 h. The red suspension was concentrated i*n vacuo* and thoroughly dried to provide a dark red solid.

The solid was suspended in CH_2Cl_2 (8 mL), and pyridine (346 μL, 4.283 mmol, 4.0 eq) and trifluoroacetic anhydride (595 μL, 4.283 mmol, 4.0 eq) were added. The reaction was stirred at room temperature for 3 h. The yellow-orange solution was diluted with 10% w/v aqueous citric acid and extracted with CH_2Cl_2 (2×). The combined organic layers were dried ($MgSO_4$), filtered, and evaporated to an orange gum. Silica gel chromatography (0→30% EtOAc/toluene, linear gradient) afforded 632 mg (90%) of **12** as a white solid.

Data for **12**: $\mathbf{R}_f$ = 0.30 (8:1 v/v toluene/EtOAc); **^{1}H NMR** (400 MHz, DMSO-d$_6$, δ): 5.44 (s, 2H), 6.97 (d, *J* = 8.7 Hz, 2H), 7.47–7.36 (m, 5H), 7.55–7.47 (m, 3H), 7.86 (d, *J* = 2.1 Hz, 2H), 8.35 (dd, *J* = 8.1, 1.5 Hz, 1H), 8.48 (dd, *J* = 1.4, 0.6 Hz, 1H), 11.54 (s, 2H); **^{13}C NMR** (100 MHz, DMSO-d$_6$, δ): 66.9 (CH_2), 81.3 (C), 108.6 (CH), 114.7 (C), 115.5 (q, $^1J_{CF}$ = 288.6 Hz, C), 117.1 (CH), 124.8 (CH), 125.9 (CH), 126.3 (C), 128.2 (CH), 128.3 (CH), 128.6 (CH), 129.1 (CH), 131.9 (C), 135.7 (C), 136.3 (CH), 138.8 (C), 150.4 (C), 154.8 (q, $^2J_{CF}$ = 37.4 Hz, C), 156.2 (C), 164.3 (C), 167.5 (C); **^{19}F NMR** (376 MHz, DMSO-d$_6$, δ): –73.6 (s); **HRMS** (*m/z*): $[M+H]^+$ calculated, 657.1091; observed, 657.1079.

Bis(2,4-dimethoxybenzyl) 4-(3,6-bis(2,2,2-trifluoroacetamido)-9H-xanthen-9-yl)isophthalate (13): To a solution of **12** (150 mg, 0.228 mmol) in THF (6 mL) was added 10% w/w palladium on carbon (61 mg). The reaction was flushed with nitrogen (3×), followed by hydrogen (3×). The reaction was stirred at room temperature for 18 h under hydrogen (balloon, 1 atm). The crude reaction was filtered through celite with EtOAc and concentrated *in vacuo* to provide a colorless solid that turned pale pink upon prolonged exposure to air.

The crude reduced rhodamine intermediate was dissolved in 1:1 v/v EtOAc/CH_2Cl_2 (10 mL). 2,4-Dimethoxybenzyl alcohol (168 mg, 1.00 mmol, 4.4 eq), EDCI (192 mg, 1.00 mmol, 4.4 eq), and DMAP (0.6 mg, 4.6 μmol, 0.02 eq) were added. The reaction was stirred at room temperature for 18 h. Silica gel was added, and the mixture was concentrated to dryness. Purification by flash chromatography (dry load with silica gel, 0→25% EtOAc/hexanes, linear gradient, with constant 40% v/v CH_2Cl_2 additive) afforded **13** as a colorless foam (128 mg, 65%).

Data for **13**: $\mathbf{R}_f$ = 0.47 (4:2:4 v/v/v hexanes/EtOAc/CH_2Cl_2); **^{1}H NMR** (400 MHz, $CDCl_3$, δ): 3.68 (s, 3H), 3.76 (s, 3H), 3.79 (s, 3H), 3.80 (s, 3H), 5.28 (s, 2H), 5.38 (s, 2H), 6.25 (s, 1H), 6.40 (d, *J* = 2.3 Hz, 1H), 6.48–6.41 (m, 3H), 6.92 (d, *J* = 8.7 Hz, 2H), 7.07–6.97 (m, 3H), 7.25–7.22 (m, 1H), 7.30 (d, *J* = 8.3 Hz, 1H), 7.42 (d, *J* = 2.2 Hz, 2H), 7.90 (dd, *J* = 8.3, 1.9 Hz, 1H), 8.16 (s, 2H), 8.44 (d, *J* = 1.8 Hz, 1H); **^{13}C NMR** (100 MHz, $CDCl_3$, δ): 38.2 (CH), 55.46 (CH_3), 55.53 (CH_3), 55.55 (CH_3), 55.59 (CH_3), 62.7 (CH_2), 63.8 (CH_2), 98.7 (CH), 104.2 (CH), 109.0 (CH), 115.8 (q, $^1J_{CF}$ = 288.6 Hz, C), 115.9 (CH), 116.1 (C), 116.6 (C), 121.8 (C), 129.1 (C), 130.5 (C), 130.7 (CH), 131.3 (CH), 131.4 (CH), 131.8 (CH), 132.0 (CH), 133.2 (CH), 135.1 (C), 150.8 (C), 151.0 (C), 155.0 (q, $^2J_{CF}$ = 37.4 Hz, C), 159.2 (C), 159.4 (C), 161.5 (C), 161.8 (C), 165.7 (C), 168.0 (C); **^{19}F NMR** (376 MHz, $CDCl_3$, δ): –76.2 (s); **HRMS** (*m/z*): $[M+Na]^+$ calculated, 891.1959; observed, 891.1947.

Bis(2,4-dimethoxybenzyl) 4-(3,6-bis((((4,5-dimethoxy-2-nitrobenzyl)oxy)carbonyl)amino)-9H-xanthen-9-yl)isophthalate (14): To a solution of diester **13** (125 mg, 0.144 mmol) in MeOH (5 mL) was added hydroxylamine (50% w/w in water, 220 μL, 3.597 mmol, 25 eq). The reaction was stirred for 24 h at room temperature while shielded from light. It was then diluted with saturated $NaHCO_3$ and extracted with EtOAc (2×). The combined organic extracts were washed with brine, dried ($MgSO_4$), filtered, and concentrated *in vacuo*. Purification by flash chromatography (25→100% EtOAc/hexanes, linear gradient) afforded 68 mg (70%) of dianiline intermediate that was used immediately.

The dianiline (68 mg, 0.100 mmol) was dissolved in CH_2Cl_2 (3 mL). 4,5-Dimethoxy-2-nitrobenzyl chloroformate (**6**, 61 mg, 0.220 mmol, 2.2 eq) in CH_2Cl_2 (1 mL) was added, followed by DIEA (44 μL, 0.250 mmol, 2.5 eq). The reaction was stirred for 18 h at room temperature while shielded from light. It was then diluted with 10% w/v aqueous citric acid and extracted with EtOAc (2×). The combined organic layers were washed with brine, dried ($MgSO_4$), filtered, and concentrated *in vacuo*. Flash chromatography on silica gel (10→50% EtOAc/toluene, linear gradient) afforded **14** as a pale yellow solid (100 mg, 87%).

Data for **14**: $\mathbf{R}_f$ = 0.37 (7:3 v/v toluene/EtOAc); **^{1}H NMR** (400 MHz, $CDCl_3$, δ): 3.67 (s, 3H), 3.76 (s, 3H), 3.79 (s, 3H), 3.80 (s, 3H), 3.94 (s, 12H), 5.27 (s, 2H), 5.39 (s, 2H), 5.57 (s, 4H), 6.19 (s, 1H), 6.40 (d, *J* = 2.3 Hz, 1H), 6.47–6.41 (m, 3H), 6.83 (dd, *J* = 8.4, 1.9 Hz, 2H), 6.87 (d, *J* = 8.5 Hz, 2H), 6.96 (s, 2H), 7.02 (s, 2H), 7.05 (d, *J* = 8.3 Hz, 1H), 7.28–7.21 (m, 3H), 7.31 (d, *J* = 8.3 Hz, 1H), 7.69 (s, 2H), 7.88 (dd, *J* = 8.3, 1.9 Hz, 1H), 8.40 (d, *J* = 1.8 Hz, 1H); **^{13}C NMR** (100 MHz, $CDCl_3$, δ): 38.0 (CH), 55.45 (CH_3), 55.51 (CH_3), 55.52 (CH_3), 55.57 (CH_3), 56.51 (CH_3), 56.59 (CH_3), 62.6 (CH_2), 63.6 (CH_2), 64.1 (CH_2), 98.7 (CH), 104.15 (CH), 104.18 (CH), 106.9 (CH), 108.4 (CH), 110.8 (CH), 114.2 (CH), 116.2 (C), 116.6 (C), 119.3 (C), 127.3 (C), 128.7 (C), 130.4 (C), 130.5 (CH), 131.0 (CH), 131.3 (CH), 131.7 (CH), 131.9 (CH), 133.0 (CH), 137.5 (C), 140.1 (C), 148.5 (C), 151.1 (C), 151.9 (C), 152.9 (C), 153.7 (C), 159.1 (C), 159.3 (C), 161.4 (C), 161.8 (C), 165.7 (C), 168.0 (C); **HRMS** (*m/z*): $[M+Na]^+$ calculated, 1177.3212; observed, 1177.3173.

3',6'-Bis((((4,5-dimethoxy-2-nitrobenzyl)oxy)carbonyl)amino)-3-oxo-3H-spiro[isobenzofuran-1,9'-xanthene]-5-carboxylic acid (15; *i.e.*, NVOC$_2$-5-carboxy-rhodamine 110): To a solution of diester **14** (66 mg, 0.0571 mmol) in CH_2Cl_2 (9 mL) and pH 7 buffer (1 mL) was added 2,3-dichloro-5,6-dicyano-1,4-benzoquinone (130 mg, 0.571 mmol, 10 eq). The reaction was heated to reflux and stirred vigorously for 24 h while being shielded from light. It was subsequently cooled to room temperature and concentrated *in vacuo*. The residue was taken up in MeOH, silica gel was added, and the mixture was again evaporated to dryness. Silica gel chromatography (dry load with silica gel, 0→5% MeOH/CH_2Cl_2, linear gradient, with constant 1% v/v AcOH additive) followed by reverse phase HPLC (35→95% CH_3CN/H_2O, linear gradient, with constant 0.1% v/v TFA additive) afforded 30 mg (62%) of **15** as a tan solid.

Data for **15**: $\mathbf{R}_f$ = 0.49 (9:1:0.1 v/v/v CH_2Cl_2/MeOH/AcOH); **^{1}H NMR** (400 MHz, DMSO-d$_6$, δ): 3.88 (s, 6H), 3.92 (s, 6H), 5.48 (s, 4H), 6.81 (d, *J* = 8.7 Hz, 2H), 7.17 (dd, *J* = 8.8, 2.1 Hz, 2H), 7.30 (s, 2H), 7.42 (dd, *J* = 8.1, 0.6 Hz, 1H), 7.60 (d, *J* = 2.0 Hz, 2H), 7.73 (s, 2H), 8.29 (dd, *J* = 8.0, 1.5 Hz, 1H), 8.42 (dd, *J* = 1.5, 0.7 Hz, 1H), 10.21 (s, 2H), 13.55 (s, 1H); **^{13}C NMR** (100 MHz, DMSO-d$_6$, δ): 56.2 (CH_3), 56.3 (CH_3), 63.2 (CH_2), 82.2 (C), 105.3 (CH), 108.3 (CH), 111.8 (CH), 112.0 (C), 114.7 (CH), 124.5 (CH), 125.6 (CH), 126.36 (C), 126.42 (C), 128.8 (CH), 133.0 (C), 136.2 (CH), 139.8 (C), 141.4 (C), 148.1 (C), 150.9 (C), 152.9 (C), 153.2 (C), 156.0 (C),

166.0 (C), 167.8 (C); **HRMS** (*m/z*): $[M+H]^+$ calculated, 853.1835; observed, 853.1836.

Bis(2,4-dimethoxybenzyl) 4-(3,6-diacetoxy-2,7-difluoro-9*H*-xanthen-9-yl)isophthalate (17). To a solution of 5-carboxy-2′,7′-difluorofluorescein diacetate **16** (250. mg, 0.504 mmol) in 12 mL EtOAc was added 10% w/w palladium on carbon (25 mg). The reaction flask was flushed thoroughly with nitrogen gas, followed by hydrogen gas. The reaction was stirred for 16 h at room temperature under 1 atm of hydrogen gas (balloon), after which time, the reaction flask was thoroughly purged with nitrogen gas. The crude reaction mixture was filtered though celite, rinsed with EtOAc (100 mL), and concentrated *in vacuo*, affording dihydrodifluorofluorescein diacetate (238.7 mg, 95% yield) as a white solid.

Data for dihydrodifluorofluorescein diacetate: **R_f** = 0.32 (9:1:0.1 v/v/v CH_2Cl_2/MeOH/AcOH); **^{1}H NMR** (400 MHz, acetone-d_6, δ): 2.29 (s, 6H), 6.71 (s, 1H), 7.09 (d, $^{4}J_{HF}$ = 6.5 Hz, 2H), 7.09 (d, $^{3}J_{HF}$ = 10.5 Hz, 2H), 7.27 (d, J = 8.1 Hz, 1H), 8.07 (dd, J = 8.1, 1.7 Hz, 1H), 8.66 (d, J = 1.7 Hz, 1H); **^{13}C NMR** (100 MHz, acetone-d_6, δ): 20.3 (CH_3), 38.9 (CH), 113.1 (br, CH), 117.3 (d, $^{2}J_{CF}$ = 21.3 Hz, CH), 122.6 (d, $^{3}J_{CF}$ = 6.5 Hz, C), 130.3 (C), 131.0 (C), 132.6 (CH), 132.7 (CH), 134.2 (CH), 138.7 (d, $^{2}J_{CF}$ = 14.6 Hz, C), 147.4 (d, $^{4}J_{CF}$ = 2.2 Hz, C), 151.2 (d, $^{1}J_{CF}$ = 244 Hz, C), 151.8 (C), 166.5 (C), 168.5 (C), 169.0 (C); **^{19}F NMR** (376 MHz, acetone-d_6, δ): –134.7 (dd, J_{FH} = 10.5, 6.5 Hz); **HRMS** (*m/z*): $[M+Na]^+$ calculated, 521.0655; observed, 521.0640.

To a solution of dihydrodifluorofluorescein diacetate (93.8 mg, 0.188 mmol) in 2 mL CH_2Cl_2 was added *N,N′*-diisopropylcarbodiimide (DIC, 128.2 µL, 0.828 mmol, 4.4 eq), 4-dimethylaminopyridine (DMAP, 0.5 mg, 3.8 µmol, 0.02 eq), and 2,4-dimethoxybenzyl alcohol (**4**, 139.3 mg, 0.828 mmol, 4.4 eq). The reaction was stirred at room temperature for 18 h, and then filtered to remove the resulting white precipitate. The filtrate was concentrated *in vacuo* and purified by column chromatography (4:1 v/v hexanes/EtOAc→3:7 v/v hexanes/EtOAc, linear gradient), affording 99.7 mg of pure tetraester **17** (66% yield). Data for **17**: **R_f** = 0.41 (1:1 v/v hexanes/EtOAc); **^{1}H NMR** (400 MHz, $CDCl_3$, δ): 2.31 (s, 6H), 3.70 (s, 3H), 3.79 (s, 3H), 3.81 (s, 3H), 3.81 (s, 3H), 5.30 (s, 2H), 5.41 (s, 2H), 6.23 (s, 1H), 6.42-6.48 (series of m, 4H), 6.75 (d, $^{3}J_{HF}$ = 10.3 Hz, 2H), 6.90 (d, $^{4}J_{HF}$ = 6.5 Hz, 2H), 7.11 (d, J = 8.3 Hz, 1H), 7.27 (d, J = 8.9 Hz, 1H), 7.34 (d, J = 8.3 Hz, 1H), 7.98 (dd, J = 8.3, 1.9 Hz, 1H), 8.46 (d, J = 1.9 Hz, 1H); **^{13}C NMR** (100 MHz, $CDCl_3$, δ): 20.5 (CH_3), 38.4 (CH), 55.3 (CH_3), 55.4 (CH_3), 55.4 (CH_3), 55.5 (CH_3), 62.5 (CH_2), 63.7 (CH_2), 98.6 (CH), 98.6 (CH), 104.0 (CH), 104.1 (CH), 111.9 (br, CH), 115.9 (C), 116.5 (C), 116.7 (d, $^{2}J_{CF}$ = 21.2 Hz, CH), 121.3 (d, $^{3}J_{CF}$ = 6.6 Hz, C), 129.4 (C), 130.4 (C), 131.2 (CH), 131.3 (CH), 131.6 (CH), 132.0 (CH), 133.3 (CH), 137.5 (d, $^{2}J_{CF}$ = 14.7 Hz, C), 146.3 (d, $^{4}J_{CF}$ = 2.2 Hz, C), 149.6 (C), 150.1 (d, $^{1}J_{CF}$ = 246 Hz, C), 159.1 (C), 159.3 (C), 161.3 (C), 161.8 (C), 165.3 (C), 167.5 (C), 168.0 (C); **^{19}F NMR** (376 MHz, $CDCl_3$, δ): –134.4 (dd, J_{FH} = 10.2, 6.0 Hz); **HRMS** (*m/z*): $[M+Na]^+$ calculated, 821.2016; observed, 821.2002.

Bis(2,4-dimethoxybenzyl) 4-(2,7-difluoro-3,6-dihydroxy-9*H*-xanthen-9-yl)isophthalate (18): To a solution of tetraester **17** (91.3 mg, 0.114 mmol) in 4 mL THF and 2 mL MeOH was added ammonium bicarbonate (90.3 mg, 1.14 mmol, 10 eq) as a solution in 2 mL H_2O. The reaction was stirred vigorously (~600 RPM) at room temperature for 24 h while shielded from light. The organic solvents were concentrated *in vacuo*, The pH was adjusted to ~3 with 10% w/v aqueous citric acid. The aqueous layer was diluted to 30 mL with H_2O and extracted with EtOAc (3×30 mL). The organic layers were combined, dried over anhydrous $MgSO_4$(s), filtered, and concentrated *in vacuo*. Purification by column chromatography (7:3 v/v hexanes/EtOAc→1:4 v/v hexanes/EtOAc, linear gradient) afforded 80.0 mg of pure diphenol **18** (98% yield).

Data for **18**: **R_f** = 0.35 (4:4:2 v/v/v hexanes/EtOAc/CH_2Cl_2); **^{1}H NMR** (400 MHz, $CDCl_3$, δ): 3.71 (s, 3H), 3.78 (s, 3H), 3.81 (s, 3H), 3.81 (s, 3H), 5.29 (s, 2H), 5.31 (br s, 2H), 5.41 (s, 2H), 6.05 (s, 1H), 6.45 (m, 4H), 6.66 (d, $^{3}J_{HF}$ = 10.8 Hz, 2H), 6.72 (d, $^{4}J_{HF}$ = 7.7 Hz, 2H), 7.07 (d, J = 8.4 Hz, 1H), 7.25 (d, J = 8.8 Hz, 1H), 7.34 (d, J = 8.2 Hz, 1H), 7.93 (dd, J = 8.2, 1.9 Hz, 1H), 8.42 (dd, J = 1.9, 0.3 Hz, 1H); **^{13}C NMR** (100 MHz, $CDCl_3$, δ): 37.9 (CH), 55.3 (CH_3), 55.4 (CH_3), 55.4 (CH_3), 55.5 (CH_3), 62.5 (CH_2), 63.6 (CH_2), 98.6 (CH), 98.6 (CH), 104.0 (CH), 104.1 (CH), 105.0 (d, $^{3}J_{CF}$ = 0.7 Hz, CH), 115.1 (d, $^{3}J_{CF}$ = 6.2 Hz, C), 115.5 (d, $^{2}J_{CF}$ = 20.2 Hz, CH), 116.0 (C), 116.5 (C), 128.9 (C), 130.1 (C), 131.0 (CH), 131.3 (CH), 131.3 (CH), 131.9 (CH), 133.0 (CH), 143.3 (d, $^{2}J_{CF}$ = 16.2 Hz, C), 146.9 (d, $^{4}J_{CF}$ = 2.1 Hz, C), 147.3 (d, $^{1}J_{CF}$ = 234 Hz, C), 151.2 (C), 159.0 (C), 159.3 (C), 161.3 (C), 161.7 (C), 165.6 (C), 167.8 (C); **^{19}F NMR** (376 MHz, $CDCl_3$, δ): –146.3 (dd, J_{FH} = 10.8, 7.7 Hz); **HRMS** (*m/z*): $[M-H]^-$ calculated, 713.1840; observed, 713.1866.

Bis(2,4-dimethoxybenzyl) 4-(3,6-bis((4,5-dimethoxy-2-nitrobenzyl)oxy)-2,7-difluoro-9*H*-xanthen-9-yl)isophthalate (20): To a solution of diphenol **18** (28.8 mg, 0.0403 mmol) in 2 mL CH_2Cl_2 and 2 mL H_2O was added tetrabutylammonium hydrogensulfate (27.4 mg, 0.0806 mmol, 2 eq), potassium carbonate (33.4 mg, 0.242 mmol, 6 eq), and 4,5-dimethoxy-2-nitrobenzyl bromide (**19**, 66.8 mg, 0.242 mmol, 6 eq). The reaction was stirred vigorously (~600 RPM) for 18 h at room temperature while shielded from light. The aqueous layer was diluted to 20 mL H_2O and was extracted with CH_2Cl_2 (3 × 45 mL). The organic layers were combined, dried over anhydrous $MgSO_4$(s), filtered, and concentrated *in vacuo*. Purification by column chromatography (4:1 v/v hexanes/EtOAc→7:13 v/v hexanes/EtOAc, linear gradient) afforded pure diether **20** as a white solid (34.7 mg, 78% yield).

Data for **20**: **R_f** = 0.20 (1:1 v/v hexanes/EtOAc); **^{1}H NMR** (400 MHz, CD_2Cl_2, δ): 3.73 (s, 3H), 3.78 (s, 3H), 3.80 (s, 3H), 3.80 (s, 3H), 3.92 (s, 6H), 3.93 (s, 6H), 5.27 (s, 2H), 5.40 (s, 2H), 5.52 (s, 4H), 6.15 (s, 1H), 6.48 (m, 4H), 6.75 (d, $^{3}J_{HF}$ = 11.4 Hz, 2H), 6.81 (d, $^{4}J_{HF}$ = 7.3 Hz, 2H), 7.11 (d, J = 8.3 Hz, 1H), 7.25 (d, J = 8.2 Hz, 1H), 7.34 (d, J = 7.1 Hz, 1H), 7.35 (s, 2H), 7.76 (s, 2H), 7.93 (dd, J = 8.3, 1.9 Hz, 1H), 8.39 (d, J = 1.9 Hz, 1H); **^{13}C NMR** (100 MHz, CD_2Cl_2, δ): 38.4 (CH), 55.7 (CH_3), 55.9 (CH_3), 56.7 (CH_3), 56.7 (CH_3), 62.8 (CH_2), 63.9 (CH_2), 68.9 (CH_2), 98.8 (CH), 98.9 (CH), 104.1 (d, $^{3}J_{CF}$ = 1.1 Hz, CH), 104.5 (CH), 104.5 (CH), 108.6 (CH), 109.9 (CH), 116.2 (d, $^{3}J_{CF}$ = 6.2 Hz, C), 116.4 (C), 116.6 (d, $^{2}J_{CF}$ = 18.5 Hz, CH), 116.9 (C), 128.5 (C), 129.6 (C), 130.8 (C), 131.2 (CH), 131.5 (CH), 131.9 (CH), 132.2 (CH), 133.2 (CH), 139.5 (C), 146.4 (d, $^{2}J_{CF}$ = 12.5 Hz, C), 147.0 (d, $^{4}J_{CF}$ = 2.2 Hz, C), 148.7 (C), 149.2 (d, $^{1}J_{CF}$ = 241 Hz, C), 151.2 (C), 154.6 (C), 159.5 (C), 159.7 (C), 161.9 (C), 162.2 (C), 165.6 (C), 167.9 (C); **^{19}F NMR** (376 MHz, CD_2Cl_2, δ): –140.5 (dd, J_{FH} = 11.4, 7.3 Hz); **HRMS** (*m/z*): $[M+Na]^+$ calculated, 1127.2868; observed, 1127.2886.

3',6'-Bis((4,5-dimethoxy-2-nitrobenzyl)oxy)-2',7'-difluoro-3-oxo-3*H*-spiro[isobenzofuran-1,9'-xanthene]-5-carboxylic acid (21; *i.e.*, NV_2-5-carboxy-2′,7′-difluorofluorescein): To a solution of diether **20** (7.2 mg, 6.52 µmol) in 1.8 mL CH_2Cl_2 and 0.2 mL H_2O was added 2,3-dichloro-5,6-dicyano-1,4-benzoquinone (DDQ, 14.8 mg, 65.2 µmol, 10 eq). The reaction was heated to reflux and stirred vigorously (~600 RPM) for 24 h while shielded from light. The resulting mixture was cooled to room temperature, filtered through a pad of celite, and concentrated *in vacuo*. Purification by column chromatography (CH_2Cl_2→ 94:6 v/v CH_2Cl_2/MeOH, linear gradient, with constant 1% v/v AcOH additive, column run twice)

afforded 4.3 mg (83% yield) of pure NV_2-5-carboxy-2′,7′-difluorofluorescein **21**.

Data for **21**: $\mathbf{R}_f$ = 0.42 (9:1:0.1 v/v/v CH_2Cl_2/MeOH/AcOH); **^{1}H NMR** (400 MHz, DMSO-d_6, δ): 3.87 (s, 6H), 3.88 (s, 6H), 5.57 (s, 4H), 6.82 (d, $^3J_{HF}$ = 11.3 Hz, 2H), 7.29 (d, $^4J_{HF}$ = 7.2 Hz, 2H), 7.33 (s, 2H), 7.43 (dd, J = 8.0, 0.5 Hz, 1H), 7.73 (s, 2H), 8.29 (dd, J = 8.1, 1.4 Hz, 1H), 8.40 (dd, J = 1.4, 0.8 Hz, 1H); **^{13}C NMR** (100 MHz, DMSO-d_6, δ): 56.1 (CH_3), 56.2 (CH_3), 68.1 (CH_2), 81.6 (C), 103.4 (br, CH), 108.4 (CH), 109.4 (d, $^3J_{CF}$ = 6.2 Hz, C); 111.7 (CH), 114.1 (d, $^2J_{CF}$ = 20.9 Hz, CH), 124.2 (CH), 125.7 (C), 126.1 (CH), 126.4 (C), 133.6 (C), 136.2 (CH), 139.9 (C), 147.2 (d, $^4J_{CF}$ = 1.4 Hz, C), 148.2 (d, $^2J_{CF}$ = 12.5 Hz, C), 148.2 (C), 148.3 (d, $^1J_{CF}$ = 243 Hz, C) 153.1 (C), 154.9 (C), 166.1 (C), 167.4 (C); **^{19}F NMR** (376 MHz, DMSO-d_6, δ): –138.0 (dd, J_{FH} = 10.9, 7.4 Hz); **HRMS** (*m/z*): $[M+H]^+$ calculated, 803.1531; observed, 803.1534.

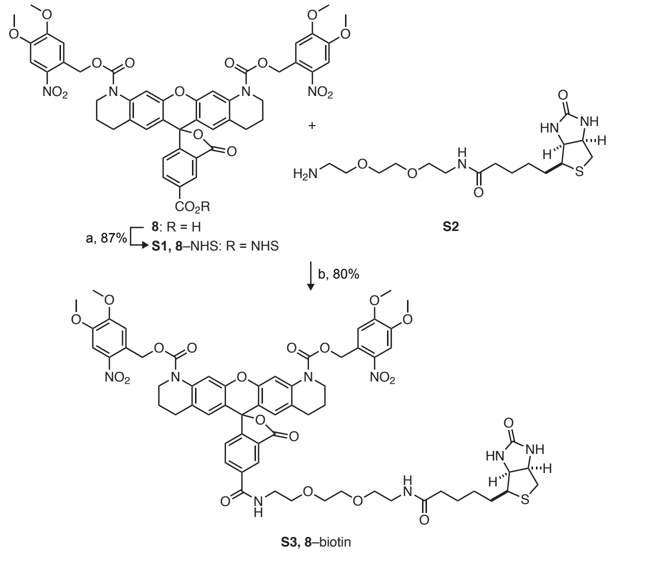

Scheme S1. Synthesis of $NVOC_2$-Rh_Q-5-PEG_2-biotin (**S3**, **8**–biotin) a) TSTU, DIEA, DMF. b) DIEA, DMF.

$NVOC_2$-5-carboxy-Q-rhodamine-NHS ester (S1; 8–NHS**)**: To a solution of acid **8** (12.1 mg, 13.0 μmol) in 0.5 mL DMF was added *N,N*-diisopropylethylamine (DIEA, 6.8 μL, 38.9 μmol, 3 eq) and *N,N,N′,N′*-tetramethyl-*O*-(*N*-succinimidyl)uronium tetrafluoroborate (TSTU, 5.1 mg, 16.9 μmol, 1.3 eq). The mixture was stirred at room temperature for 16 h, shielded from light. The reaction was partitioned between 50 mL EtOAc and 50 mL 10% w/v aqueous citric acid. The organic layer was washed with H_2O (2×50 mL) and brine (50 mL), dried over anhydrous $MgSO_4$(s), filtered, and concentrated *in vacuo*. Purification by column chromatography (CH_2Cl_2→EtOAc, linear gradient) afforded 11.6 mg (87% yield) of NHS ester **S1** (**8**–NHS) as an off-white solid.

Data for **S1**: $\mathbf{R}_f$ = 0.53 (EtOAc); **^{1}H NMR** (400 MHz, $CDCl_3$, δ): 1.91 (m, 4H), 2.58 (ABXY, J_{AB} = 16.1 Hz, J_{AX} = 6.5 Hz, J_{AY} = 6.5 Hz, 2H), 2.64 (ABXY, J_{AB} = 16.1 Hz, J_{BX} = 6.4 Hz, J_{BY} = 6.4 Hz, 2H), 2.96 (br s, 4H), 3.79 (ABXY, J_{AB} = 12.6 Hz, J_{AX} = 6.1 Hz, J_{AY} = 6.1 Hz, 2H), 3.83 (ABXY, J_{AB} = 12.6 Hz, J_{BX} = 6.1 Hz, J_{BY} = 6.1 Hz, 2H), 3.91 (s, 6H), 3.96 (s, 6H), 5.66 (ABq, J_{AB} = 14.6 Hz, 2H), 5.69 (ABq, J_{AB} = 14.6 Hz, 2H), 6.47 (s, 2H), 7.04 (s, 2H), 7.31 (dd, J = 8.1, 0.7 Hz, 1H), 7.73 (s, 2H), 7.80 (br s, 2H), 8.40 (dd, J = 8.1, 1.5 Hz, 1H), 8.82 (dd, J = 1.5, 0.7 Hz, 1H); **^{13}C NMR** (100 MHz, $CDCl_3$, δ): 22.9 (CH_2), 25.7 (CH_2), 26.9 (CH_2), 45.2 (CH_2), 56.3 (CH_3), 56.4 (CH_3), 65.0 (CH_2), 82.9 (C), 108.3 (CH), 110.7 (CH), 111.6 (CH), 112.7 (C), 124.9 (CH), 126.3 (C), 127.0 (C), 127.1 (CH), 127.2 (C), 127.3 (C), 128.0 (CH), 136.8 (CH), 140.1 (C), 140.2 (C), 148.3 (C), 149.4 (C), 153.5 (C), 153.9 (C), 158.7 (C), 160.5 (C), 167.7 (C), 168.9 (C); **HRMS** (*m/z*): $[M+Na]^+$ calculated, 1052.2444; observed, 1052.2434.

$NVOC_2$-Q-rhodamine-5-PEG_2-biotin (S3, **8**–biotin**)**: To a solution of NHS ester **S1** (**8**–NHS; 2.0 mg, 1.94 μmol) in 200 μL DMF was added *N,N*-diisopropylethylamine (DIEA, 1.7 μL, 9.71 μmol, 5.0 eq) and biotinylated amine **S2** (1.1 mg, 2.91 μmol, 1.5 eq). The reaction was shielded from light and stirred at room temperature for 18 h. The mixture was concentrated *in vacuo* and purified by preparative reverse-phase HPLC (1:1 v/v CH_3CN/H_2O→95:5 v/v CH_3CN/H_2O, linear gradient, with no TFA additive, R_T = 7.9 min) to afford the biotinylated caged dye **S3** as a pure white solid (2.0 mg, 80.% yield).

Data for **S3**: $\mathbf{R}_f$ = 0.45 (9:1:0.1 v/v/v CH_2Cl_2/MeOH/AcOH); **^{1}H NMR** (400 MHz, $CDCl_3$, δ): 1.45 (m, 2H), 1.70 (m, 4H), 1.90 (m, 4H), 2.24 (t, J = 6.7 Hz, 2H), 2.56 (ABXY, J_{AB} = 16.1 Hz, J_{AX} = 6.6 Hz, J_{AY} = 6.6 Hz, 2H), 2.63 (ABXY, J_{AB} = 16.1 Hz, J_{BX} = 6.4 Hz, J_{BY} = 3.8 Hz, 2H), 2.76 (d, J = 12.9 Hz, 1H), 2.91 (dd, J = 12.9, 4.9 Hz, 1H), 3.17 (td, J = 7.2, 4.6 Hz, 1H), 3.40 (m, 2H), 3.86-3.55 (series of m, 14H), 3.90 (s, 6H), 3.96 (s, 6H), 4.33 (dd, J = 7.4, 4.9 Hz, 1H), 4.55 (dd, J = 7.4, 4.9 Hz, 1H), 5.38 (br s, 1H), 5.66 (ABq, J_{AB} = 14.7 Hz, 2H), 5.69 (ABq, J_{AB} = 14.7 Hz, 2H), 6.46 (s, 2H), 6.54 (br t, J = 5.0 Hz, 1H), 7.03 (s, 2H), 7.23 (d, J = 8.0 Hz, 1H), 7.31 (m, 1H), 7.73 (s, 2H), 7.77 (br s, 2H), 8.12 (br t, J = 4.6 Hz, 1H), 8.35 (dd, J = 8.2, 1.1 Hz, 1H), 8.66 (s, 1H); **LC–MS**: 1:1 v/v CH_3CN/H_2O→95:5 v/v CH_3CN/H_2O, linear gradient, with constant 0.1% v/v TFA additive, R_T = 7.7 min, 98% purity, mass spec signal $[M + H]^+$ calculated, 1289.4; observed, 1289.1; **HRMS** (*m/z*): $[M+Na]^+$ calculated, 1311.4163; observed, 1311.4153.

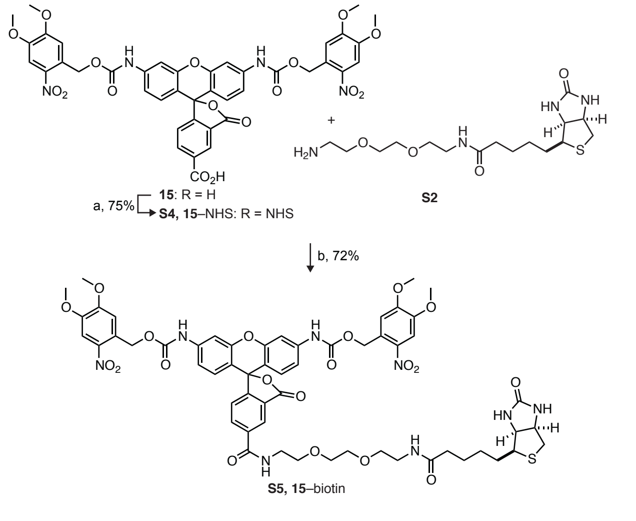

Scheme S2. Synthesis of $NVOC_2$-Rh_{110}-5-PEG_2-biotin (**S5**, **15**–biotin) a) TSTU, DIEA, DMF. b) DIEA, DMF.

$NVOC_2$-5-carboxy-rhodamine 110-NHS ester (S4, 15–NHS**):** To a solution of acid **15** (25 mg, 0.0293 mmol) and TSTU (11.5 mg, 0.0382 mmol, 1.3 eq) in DMF (2 mL) was added DIEA (15.4 μL, 0.0879 mmol, 3.0 eq). The reaction was stirred at room temperature for 18 h while being shielded from light. It was subsequently diluted with 10% w/v citric acid and extracted with EtOAc (2×). The combined organic extracts were washed with brine, dried ($MgSO_4$), filtered, and concentrated *in vacuo*. Silica gel

chromatography (10→100% EtOAc/CH_2Cl_2, linear gradient) yielded 21 mg (75%) of **S4** as an off-white solid.

Data for **S4**: **R*f*** = 0.55 (2:1 v/v CH_2Cl_2/EtOAc); **^{1}H NMR** (400 MHz, DMSO-d$_6$, δ): 2.94 (s, 4H), 3.88 (s, 6H), 3.92 (s, 6H), 5.49 (s, 4H), 6.89 (d, *J* = 8.7 Hz, 2H), 7.17 (dd, *J* = 8.8, 2.1 Hz, 2H), 7.30 (s, 2H), 7.59 (dd, *J* = 8.1, 0.4 Hz, 1H), 7.62 (d, *J* = 2.0 Hz, 2H), 7.73 (s, 2H), 8.44 (dd, *J* = 8.1, 1.6 Hz, 1H), 8.57 (dd, *J* = 1.5, 0.7 Hz, 1H), 10.22 (s, 2H); **^{13}C NMR** (100 MHz, DMSO-d$_6$, δ): 25.6 (CH_2), 56.1 (CH_3), 56.3 (CH_3), 63.2 (CH_2), 82.6 (C), 105.3 (CH), 108.3 (CH), 111.5 (C), 111.8 (CH), 114.7 (CH), 125.7 (CH), 126.3 (C), 126.5 (C), 126.8 (CH), 127.3 (C), 129.1 (CH), 136.6 (CH), 139.8 (C), 141.6 (C), 148.1 (C), 151.0 (C), 152.9 (C), 153.2 (C), 157.8 (C), 160.8 (C), 167.1 (C), 170.1 (C); **HRMS** (*m/z*): $[M+H]^+$ calculated, 950.1999; observed, 950.2000.

NVOC$_2$-rhodamine 110-5-PEG$_2$-biotin (S5, 15–biotin**):** Ester **S4** (10 mg, 0.0105 mmol) was dissolved in DMF (1 mL). Biotinylated amine **S2** (5.9 mg, 0.0158 mmol, 1.5 eq) and DIEA (9.2 μL, 0.0526 mmol, 5.0 eq) were added, and the reaction was stirred at room temperature for 18 h while being shielded from light. The reaction was concentrated *in vacuo* and purified by reverse phase HPLC (30→95% CH_3CN/H_2O, linear gradient, with no additive) to provide **S5** (9.2 mg, 72%) as an off-white solid.

Data for **S5**: **R*f*** = 0.44 (9:1:0.1 v/v/v CH_2Cl_2/MeOH/AcOH); **^{1}H NMR** (400 MHz, DMSO-d$_6$, δ): 1.66–1.19 (m, 7H), 2.05 (t, *J* = 7.4 Hz, 2H), 2.57 (d, *J* = 12.3 Hz, 1H), 2.80 (dd, *J* = 12.4, 5.1 Hz, 1H), 3.12–3.02 (m, 1H), 3.17 (q, *J* = 5.9 Hz, 2H), 3.40 (t, *J* = 5.9 Hz, 2H), 3.62–3.44 (m, 7H), 3.88 (s, 6H), 3.92 (s, 6H), 4.14–4.07 (m, 1H), 5.48 (s, 4H), 4.33–4.24 (m, 1H), 6.36 (d, *J* = 22.3 Hz, 2H), 6.78 (d, *J* = 8.7 Hz, 2H), 7.17 (dd, *J* = 8.7, 2.1 Hz, 2H), 7.30 (s, 2H), 7.40 (d, *J* = 7.9 Hz, 1H), 7.60 (d, *J* = 2.0 Hz, 2H), 7.73 (s, 2H), 7.80 (t, *J* = 5.8 Hz, 1H), 8.25 (dd, *J* = 8.1, 1.5 Hz, 1H), 8.50 (s, 1H), 8.90 (t, *J* = 5.4 Hz, 1H), 10.22 (s, 2H); **LC–MS**: 30→95% CH_3CN/H_2O, linear gradient, with constant 0.1% v/v TFA additive, R_T = 12.1 min, 98% purity, mass spec signal $[M+H]^+$ calculated, 1209.4; observed, 1209.2; **HRMS** (*m/z*): $[M+H]^+$ calculated, 1209.3717; observed, 1209.3750.

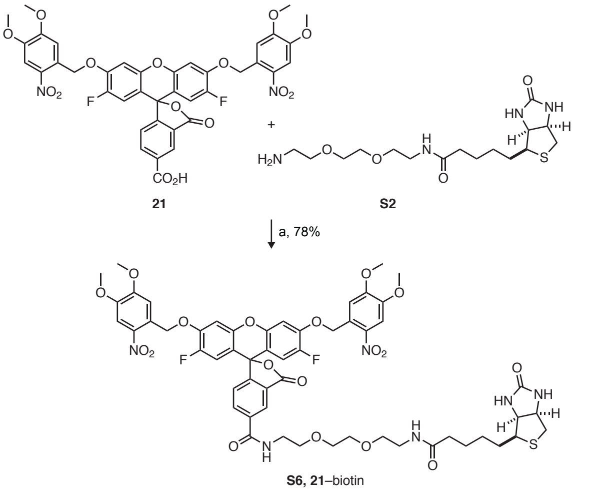

Scheme S3. Synthesis of NV$_2$-2′,7′-difluorofluorescein-5-PEG$_2$-biotin (**S6; 21**–biotin): a) TSTU, DIEA, DMF.

NV$_2$-2′,7′-difluorofluorescein-5-PEG$_2$-biotin (S6; **21**–biotin**)**: To a solution of acid **21** (5.0 mg, 6.23 μmol) in 0.5 mL DMF was added *N,N*-diisopropylethylamine (DIEA, 21.7 μL, 0.125 mmol, 20 eq) and *N,N,N′,N′*-tetramethyl-*O*-(*N*-succinimidyl)uronium tetrafluoroborate (TSTU, 2.4 mg, 8.10 μmol, 1.3 eq). The reaction was shielded from light and stirred at room temperature for 1.5 h. Biotinylated amine **S2** (7.0 mg, 18.7 μmol, 3.0 eq) was added, and the mixture stirred an additional 16 h. The reaction was concentrated *in vacuo* and purified by preparative reverse-phase HPLC (1:1 v/v CH_3CN/H_2O→95:5 v/v CH_3CN/H_2O, gradient, with constant 0.1% v/v TFA additive, R_T = 6.4 min) to afford the biotinylated caged dye **S6** as a pure solid (5.6 mg, 78% yield).

Data for **S6**: **R*f*** = 0.35 (9:1:0.1 v/v/v CH_2Cl_2/MeOH/AcOH); **^{1}H NMR** (400 MHz, DMSO-*d*$_6$, δ): 1.26 (m, 2H), 1.46 (m, 3H), 1.58 (m, 1H), 2.04 (t, *J* = 7.4 Hz, 2H), 2.55 (d, *J* = 12.6 Hz, 1H), 2.79 (dd, *J* = 12.6, 5.1 Hz, 1H), 3.07 (ddd, *J* = 8.3, 6.0, 4.5 Hz, 1H), 3.17 (dt, *J* = 5.8, 5.7 Hz, 2H), 3.39 (t, *J* = 5.9 Hz, 2H), 3.46-3.58 (series of m, 8H), 3.87 (s, 6H), 3.88 (s, 6H), 4.10 (ddd, *J* = 6.8, 4.5, 1.5 Hz, 1H), 4.28 (dd, *J* = 7.4, 5.3 Hz, 1H), 5.58 (s, 4H), 6.32 (s, 1H), 6.37 (s, 1H), 6.78 (d, $^3J_{HF}$ = 11.3 Hz, 2H), 7.30 (d, $^4J_{HF}$ = 7.3 Hz, 2H), 7.33 (s, 2H), 7.43 (d, *J* = 8.1 Hz, 1H), 7.74 (s, 2H), 7.79 (t, *J* = 5.6 Hz, 1H), 8.26 (dd, *J* = 8.1, 1.1 Hz, 1H), 8.49 (dd, *J* = 0.8, 0.6 Hz, 1H), 8.89 (t, *J* = 5.3 Hz, 1H); **^{13}C NMR** (100 MHz, DMSO-*d*$_6$, δ): 25.2, 28.0, 28.1, 30.6, 35.0, 38.4, 55.4, 56.1, 56.2, 59.1, 61.0, 68.1, 68.7, 69.1, 69.5, 81.5, 103.4, 108.4, 109.5 (d, $^3J_{CF}$ = 5.9 Hz), 111.7, 114.0 (d, $^2J_{CF}$ = 21.0 Hz), 124.0, 125.6, 126.2, 134.8, 136.3, 140.0, 147.2 (d, $^4J_{CF}$ = 1.5 Hz), 148.1 (d, $^2J_{CF}$ = 12.4 Hz), 148.2, 148.2 (d, $^1J_{CF}$ = 243 Hz), 153.1, 153.6, 162.6, 164.6, 167.7, 172.1; **^{19}F NMR** (376 MHz, DMSO-*d*$_6$, δ) –137.9 (dd, *J* = 10.9, 7.5 Hz); **HRMS** (*m/z*): $[M+H]^+$ calculated, 1159.3413; observed, 1159.3428.

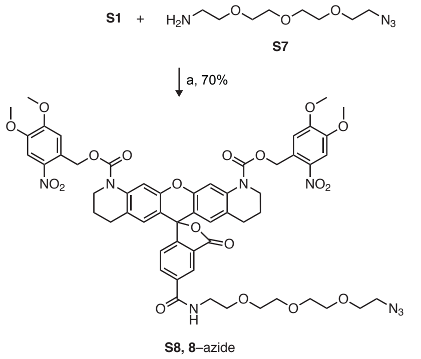

Scheme S4. Synthesis of NVOC$_2$-Rh$_Q$-5-PEG$_3$-azide (**S8**, **8**–azide) a) DIEA, DMF.

NVOC$_2$-Q-rhodamine-5-PEG$_3$-azide (S8, 8–azide): To a solution of NHS ester **S1** (3.65 mg, 3.54 μmol) in 200 μL DMF was added *N,N*-diisopropylethylamine (DIEA, 3.1 μL, 17.7 μmol, 5.0 eq) and amine **S7** (1.55 mg, 7.09 μmol, 2.0 eq). The reaction was shielded from light and stirred at room temperature for 18 h. The mixture was concentrated *in vacuo* and purified by column chromatography (1:1 v/v CH_2Cl_2/EtOAc→EtOAc, linear gradient) to afford the caged dye–azide conjugate **S8** (**8**–azide) as a pure white solid (2.8 mg, 70.% yield).

Data for **S8**: **R*f*** = 0.26 (EtOAc); **^{1}H NMR** (400 MHz, $CDCl_3$, δ): 1.90 (m, 4H), 2.55 (<u>A</u>BXY, J_{AB} = 16.1 Hz, J_{AX} = 6.6 Hz, J_{AY} = 6.6 Hz, 2H), 2.61 (A<u>B</u>XY, J_{AB} = 16.1 Hz, J_{BX} = 6.4 Hz, J_{BY} = 6.4 Hz, 2H), 3.37 (dd, *J* = 5.2, 4.7 Hz, 2H), 3.67 (dd, *J* = 5.4, 4.6 Hz, 2H), 3.71 (m, 12H), 3.77 (<u>A</u>BXY, J_{AB} = 12.8 Hz, J_{AX} = 6.2 Hz, J_{AY} = 6.2 Hz, 2H), 3.82 (A<u>B</u>XY, J_{AB} = 12.8 Hz, J_{BX} = 5.9 Hz, J_{BY} = 5.9 Hz, 2H), 3.90 (s, 6H), 3.96 (s, 6H), 5.65 (<u>A</u>Bq, J_{AB} = 14.7 Hz, 2H), 5.69 (A<u>B</u>q, J_{AB} = 14.7 Hz, 2H), 6.46 (s, 2H), 7.03 (s, 2H), 7.22 (m, 2H), 7.73 (s, 2H), 7.77 (br s, 2H), 8.21 (dd, *J* = 8.0, 1.5 Hz, 1H), 8.42 (s, 1H); **^{13}C NMR** (100 MHz, $CDCl_3$, δ): 23.0, 26.9, 29.7, 40.1,

45.2, 50.7, 56.3, 56.4, 65.0, 69.7, 70.0, 70.3, 70.5, 70.7, 70.7, 82.8, 108.3, 110.7, 111.6, 113.4, 123.4, 124.4, 126.1, 126.7, 127.1, 127.2, 134.8, 136.9, 140.0, 140.0, 148.3, 149.5, 153.5, 154.0, 155.4, 165.8, 168.7; **HRMS** (*m/z*): $[M+H]^+$ calculated, 1133.3734; observed, 1133.3731.

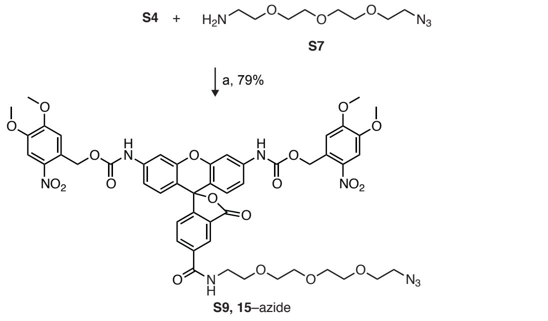

Scheme S5. Synthesis of NVOC$_2$-Rh$_{110}$-5-PEG$_3$-azide (**S9**, **15**–azide) a) DIEA, DMF.

NVOC$_2$-rhodamine 110-5-PEG$_3$-azide (S9, 15–azide): Ester **S4** (10 mg, 0.0105 mmol) was dissolved in DMF (1 mL). Amine **S7** (4.6 mg, 0.0211 mmol, 2.0 eq) and DIEA (9.1 mL, 0.0525 mmol, 5.0 eq) were added, and the reaction was stirred at room temperature for 18 h while being shielded from light. The reaction was concentrated *in vacuo* and purified by reverse phase HPLC (50→95% CH_3CN/H_2O, linear gradient, with no additive) to provide **S9** (8.8 mg, 79%) as a white solid.

Data for **S9**: $\mathbf{R_f}$ = 0.44 (EtOAc); ^{1}**H NMR** (400 MHz, DMSO-d$_6$, d): 3.40–3.33 (m, 2H), 3.52–3.44 (m, 2H), 3.63–3.52 (m, 12H), 3.88 (s, 6H), 3.92 (s, 6H), 5.48 (s, 4H), 6.77 (d, J = 8.7 Hz, 2H), 7.17 (dd, J = 8.8, 2.1 Hz, 2H), 7.30 (s, 2H), 7.40 (d, J = 8.0 Hz, 1H), 7.60 (d, J = 1.9 Hz, 2H), 7.73 (s, 2H), 8.26 (dd, J = 8.1, 1.5 Hz, 1H), 8.50 (s, 1H), 8.89 (t, J = 5.5 Hz, 1H), 10.21 (s, 2H); ^{13}**C NMR** (100 MHz, DMSO-d$_6$, d): 50.0 (CH_2), 56.1 (CH_3), 56.3 (CH_3), 63.2 (CH_2), 68.7 (CH_2), 69.2 (CH_2), 69.59 (CH_2), 69.67 (CH_2), 69.77 (CH_2), 69.79 (CH_2), 82.1 (C), 105.3 (CH), 108.3 (CH), 111.8 (CH), 112.2 (C), 114.7 (CH), 123.5 (CH), 124.2 (CH), 126.1 (C), 126.3 (C), 128.7 (CH), 134.8 (CH), 136.3 (C), 139.8 (C), 141.4 (C), 148.1 (C), 150.9 (C), 152.9 (C), 153.2 (C), 154.6 (C), 164.6 (C), 168.1 (C); **HRMS** (*m/z*): $[M+H]^+$ calculated, 1053.3108; observed, 1053.3118.

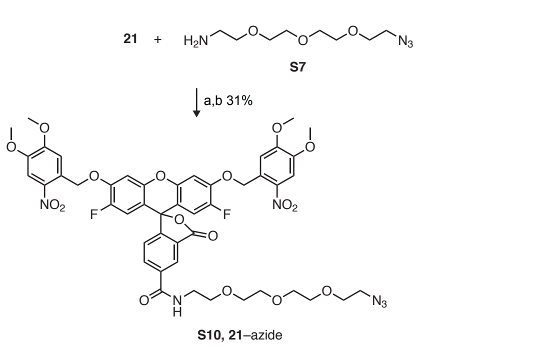

Scheme S6. Synthesis of NV$_2$-2′,7′-difluorofluorescein-5-PEG$_3$-azide **(S10; 21**–azide): a) TSTU, DIEA, DMF. b) **S7**, DIEA, DMF.

NV$_2$-2′,7′-difluorofluorescein-5-PEG$_3$-azide (S10; **21**–biotin**)**: Acid **21** (10.6 mg, 0.0132 mmol) was dissolved in DMF (0.5 mL). DIEA (6.9 µL, 0.172 mmol, 3 eq) and TSTU (5.2 mg, 0.0172 mmol, 1.3 eq) were added, and the reaction was shielded from light and stirred at room temperature for 1.5 h. The mixture was concentrated *in vacuo* and purified by column chromatography (1:1 v/v CH_2Cl_2/EtOAc→EtOAc, linear gradient) to afford the intermediate NHS ester as a white solid (7.3 mg). This material was dissolved in DMF (0.25 mL). Amine **S7** (3.5 mg, 0.0162 mmol, 2.0 eq) and DIEA (7.1 mL, 0.0406 mmol, 5.0 eq) were added, and the reaction was stirred at room temperature for 18 h while shielded from light. The reaction was concentrated *in vacuo* and purified by reverse phase HPLC (50→95% CH_3CN/H_2O, linear gradient, with no additive) to provide **S10** (4.1 mg, 31%, two steps) as a white solid.

Data for **S10**: ^{1}**H NMR** (400 MHz, $CDCl_3$, δ): 3.40–3.32 (m, 2H), 3.80–3.62 (m, 14H), 3.98 (s, 12H), 5.60 (s, 4H), 6.50 (d, J = 10.8 Hz, 2H), 6.99 (d, J = 7.0 Hz, 2H), 7.15 (s, 1H), 7.27 (m, 1H), 7.40 (s, 2H), 7.81 (s, 2H), 8.27 (dd, J = 8.0, 1.6 Hz, 1H), 8.43 (m, 1H); ^{13}**C NMR** (100 MHz, $CDCl_3$, δ): 40.19, 50.69, 56.47, 56.51, 68.32, 69.62, 70.00, 70.37, 70.57, 70.72, 70.73, 82.34, 103.48, 108.17, 109.20, 109.87 (d, $^3J_{CF}$ = 5.9 Hz), 114.04 (d, $^2J_{CF}$ = 20.9 Hz), 123.56, 124.31, 126.58, 127.72, 135.18, 137.39, 139.01, 147.80 (d, $^4J_{CF}$ = 1.6 Hz), 148.25, 148.52 (d, $^2J_{CF}$ = 12.4 Hz), 149.15 (d, $^1J_{CF}$ = 243 Hz), 154.22, 154.52, 165.51, 167.96. **HRMS** (*m/z*): $[M+Na]^+$ calculated, 1025.2623; observed, 1025.2629.

GENERAL METHODS

Preparation of Samples for Photon Yield Assessment. Rh$_Q$ **8**–biotin (**S3**), Rh$_{110}$ **15**–biotin (**S5**), and fluorescein **21**–biotin (**S6**) were dissolved in DMSO (5 mM). These stock solutions were diluted 1:1000 into phosphate-buffered saline (PBS) and filtered through a 0.5 µm syringe filter (stainless steel frit; Upchurch Scientific). 100 µL of the caged dye solutions was applied to streptavidin-coated coverslips (Xenopore), incubated for 5 min in the dark, rinsed with PBS and H_2O, and dried under a stream of N_2(g). Photoactivatable fluorescent protein mEos2 (a gift from Loren Looger, Janelia Farm Research Campus) was biotinylated using an EZ-Link Micro-PEO$_4$-Biotinylation kit from Pierce Thermo Scientific (product number 21955).

Chemical Stability of Caged Fluorophores. Buffers contained 138 mM NaCl and 10 mM each of NaOAc, NaH_2PO4, $NaHCO_3$, and Tris. The pH of these buffers was adjusted with 1.0 M HCl or 1.0 M NaOH. Rh$_Q$ **8**–biotin (**S3**), Rh$_{110}$ **15**–biotin (**S5**), and fluorescein **21**–biotin (**S6**) were dissolved in DMSO (100 µM). These stock solutions were diluted 1:100 into buffer and transferred to black, clear-bottom, 96-well polystyrene microplates from Corning (product number 3651) and sealed with sealing mats from Phenix Research Products. Plates were read from the bottom on a FlexStation 3 from Molecular Devices. Results were normalized to Rh$_Q$, Rh$_{110}$, or 2′,7′-difluorofluorescein (1 µM) in buffer and plotted as shown in Figure S1.

Cell Culture. GeneSwitch 3T3 cells (Invitrogen) were cultured in high glucose Dulbecco's Modified Eagle's Medium (DMEM) supplemented with 10% v/v newborn calf serum, 2 mM L-glutamine, 1 mM sodium pyruvate and 50 µg/mL hygromycin-B (Invitrogen) and maintained at 37 °C in a humidified 5% CO_2 v/v environment. For confocal microscopy, Lab-Tek II chambers with #1.5 borosilicate coverglass bottons (Nunc) were coated with 15 µg/mL human fibronectin (Millipore) prior to culturing of cells in DMEM supplemented with 10% v/v fetal bovine serum.

EdU Labeling and Cryosectioning. DNA labeling was accomplished using the Click-iT EdU Alexa Fluor 488 Imaging Kit (Invitrogen) replacing the Alexa Fluor 488 azide with compound **8**–azide (**S8**), **15**–azide (**S9**), or **21**–azide (**S10**). Cells were incubated

with 10 μM 5-ethynyl-2′-deoxyuridine (EdU) for 2 h at 37 °C. After EdU incubation, cells were fixed for 15 min at 37 °C with 4% w/v paraformaldehyde in 0.1 M phosphate buffer, pH 7.4. Fluorophore labeling was done per manufacturer's instructions with the following modifications: the Click-iT reaction mixture contained 4 μM of the caged dye–azide (**S8**, **S9**, or **S10**) and was filtered through a 0.5 μm steel frit syringe filter (Upchurch) prior to use; cells were permeabilized in 0.25% v/v Triton X-100 in PBS, and washed in 3% w/v bovine serum albumin BSA with 0.25% v/v Triton X-100 in PBS for all wash steps.

To ensure labeling was specific to newly synthesized DNA, EdU was omitted from the protocol or cells were pretreated with 20 μM aphidicolin (Sigma) for 1 h prior to and included in the 2 h incubation with 10 μM EdU at 37 °C to inhibit nuclear DNA polymerase alpha. Cells were then labelled with Rh_Q **8**–azide, followed by confocal imaging using a Zeiss LSM 510 META microscope equipped with a 100×/1.4 NA Plan-Apochromat objective. Rh_Q **8**–azide was uncaged using a 405 nm diode laser (30 mW). A DPSS 561 nm diode laser (15 mW) was used for excitation of the uncaged Rh_Q **8**–azide. The 405 nm activation light was applied continuously during 512 × 512 8-bit *z*-stack acquisitions using sequential scanning and 0.8 μm steps. In the control experiments no labeling was observed (data not shown).

Samples for cryosectioning were prepared using a modified Tokuyasu method protocol.[3] After incubation with EdU, cells were rinsed with warm PBS, fixed for 15 min at 37°C with 4% w/v paraformaldehyde with 0.2% w/v glutaraldehyde in 0.1 M phosphate buffer, pH 7.4 and continued for an additional 1 h at room temperature in fresh fixative. Cells were rinsed in PBS and treated with 50 mM glycine in PBS for 15 min at room temperature. Labeling of EdU with caged dye–azides **S8–S10** was done with modifications as described above. Cells were scraped in 1% w/v BSA in PBS, pelleted, embedded in 10 % w/v gelatin (porcine skin type A bloom 300; Sigma) and then infiltrated with 2.3 M sucrose in 0.1 M phosphate buffer, pH 7.4. Cryosections (150 nm) were cut on a Leica EM FC6 ultramicrotome and collected on 18 mm diameter glass cover slips for PALM imaging.

Photoactivated Localization Microscopy (PALM). Imaging was performed on instrumentation described previously.[4] Laser light was delivered to the rear pupil of a 60×/1.49 NA objective on an Olympus IX81 inverted wide field microscope. Photoactivatable fluorophores were uncaged with a 50 mW 405 nm laser (Coherent 405-50C) at 3–6 μW. Uncaged Rh_Q derivatives were excited with a 200 mW 532 nm laser (Sanctity Laser SVL-532-0200) at 6 mW power, measured at the entrance to the objective. Simultaneous excitation and activation of the sample and fluorescence emission collection was performed by placing an appropriate dichroic (Di01-R532-25x36; Semrock) and Brightline bandpass filters (FF01-582/75-25; Semrock) inside standard Olympus filter cubes. A Razor Edge long-pass filter (LP03-532RU-25; Semrock) was also inserted underneath the filter wheel to block any residual excitation light. Uncaged Rh_{110} and difluorofluorescein derivates were excited with a 488 laser (Spectra-Physics #PC14584) at 4 mW power, as above using Chroma T4951p dichroic and ET525/50 emission filters. Activated mEos2 was excited with a 561 nm laser (Crystal laser GCL-150-561) at 10 mW objective entrance power and emission collection using FF562-Di02-25x36 dichroic and FF01-617/73-25 emission filters (Semrock). Images were detected with an electron multiplying CCD camera (Andor DV887ECS-BV).

For photon yield experiments the illumination intensity for experiments using Rh_Q **8**–biotin (**S3**), Rh_{110} **15**–biotin (**S5**), and fluorescein **21**–biotin (**S6**) were approximately 1100, 425, and 525 W/cm^2 respectively. Photon yields were determined based on the electron multiplying gain and the EMCCD camera sensitivity. At typical maximum gain this value is 15.1 CCD counts per photon. Data were compiled from a sampling of 10,000 images per analysis. A pre-bleach excitation laser exposure of 2,000 frames was used to eliminate background fluorescence from these commercial slides. Molecular events were analyzed as PALM data. Histogram plots of photons per event typically had approximately 3,000 events at the peak value (Figure S2).

Single molecule frame times of 100 ms commonly yielded complete PALM images collected within 15,000 frames or approximately 25 minutes. Sample drift within this time was corrected by tracking the movement of 100 nm Au fiducial beads (Microspheres-Nanospheres 790122-010). Molecule localization and image rendering algorithms used were similar to those previously described.[5]

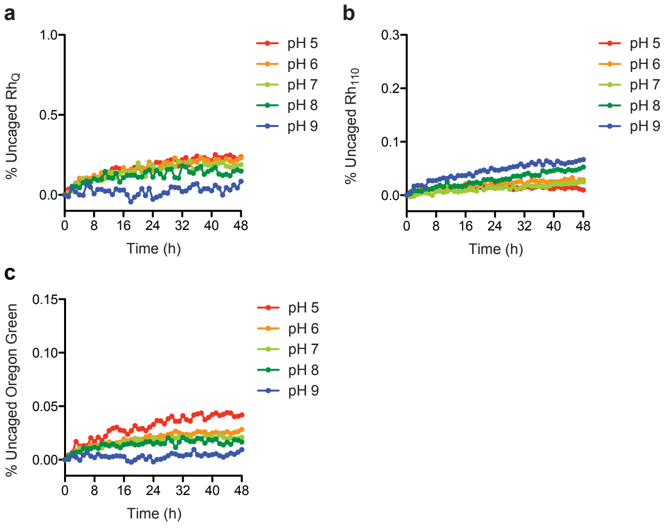

Figure S1. Stability of caged fluorophores in aqueous solution as a percentage of uncaged fluorophore *vs.* time. (a) Rh_Q **8**–biotin. (b) Rh_{110} **15**–biotin. (c) Difluorofluorescein **21**–biotin.

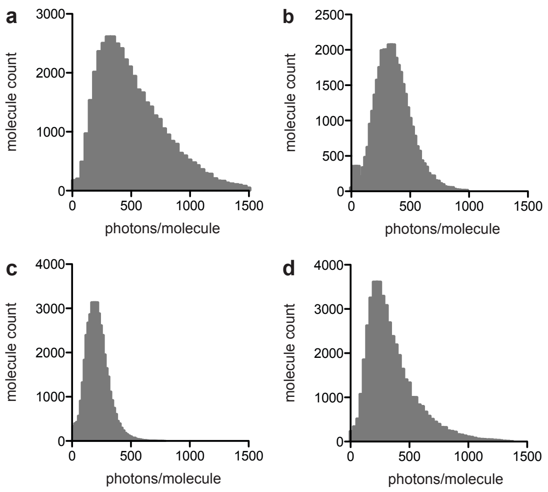

Figure S2. Representative histograms showing molecule counts against photon yields per molecule. (a) Rh_Q **8**–biotin; mean photon yield = 540, histogram peak = 324. (b) Rh_{110} **15**–biotin; mean photon yield = 377, histogram peak = 340. (c) Difluorofluorescein **21**–biotin; mean photon yield = 235, histogram peak = 192. (d) mEos2–biotin; mean photon yield = 389, histogram peak = 224.

REFERENCES

[1] W.-C. Sun, K. R. Gee, D. H. Klaubert, R. P. Haugland, *J. Org. Chem.* **1997**, *62*, 6469–6475.

[2] M. Uddin, L. Marnett, *Org. Lett.* **2008**, *10*, 4799–4801.

[3] K. Tokuyasu, *J. Cell Biol.* **1973**, *57*, 551–565.

[4] H. Shroff, C. Galbraith, J. Galbraith, H. White, J. Gillette, S. Olenych, M. Davidson, E. Betzig, *Proc. Natl. Acad. Sci. U.S.A.* **2007**, *104*, 20308–20313.

[5] E. Betzig, G. H. Patterson, R. Sougrat, O. W. Lindwasser, S. Olenych, J. S. Bonifacino, M. W. Davidson, J. Lippincott-Schwartz, H. F. Hess, *Science* **2006**, *313*, 1642–1645.
